# Multi-allelic QTL analysis of protein content in a bi-parental population of cultivated tetraploid potato

**DOI:** 10.1007/s10681-018-2331-z

**Published:** 2019-01-08

**Authors:** Michiel T. Klaassen, Peter M. Bourke, Chris Maliepaard, Luisa M. Trindade

**Affiliations:** 1 Plant Breeding, Wageningen University and Research, P.O. Box 386, 6700 AJ Wageningen, The Netherlands; 2 Department of Applied Research, Aeres University of Applied Sciences, P.O. Box 374, 8250 AJ Dronten, The Netherlands

**Keywords:** Protein content, Potato, Tetraploid, Haplotypes, Alleles, QTL analysis

## Abstract

Protein content is a key quality trait for the potato starch industry. The objective of this study was to identify allele-specific quantitative trait loci (QTLs) for tuber protein content in cultivated potato (*Solanum tuberosum* L.) at the tetraploid level. We analysed 496 full-sib F_1_ clones in a 3-year field trial to dissect the complex genetic architecture of soluble tuber protein content. Genotypic data from a 60K single nucleotide polymorphism (SNP) array was used for SNP dosage scoring, constructing homologue specific linkage maps and assembly of a dense integrated chromosomal linkage map. From the integrated map, probabilistic multi-locus identity-by-descent (IBD) haplotypes (alleles) were estimated and used to detect associations between the IBD haplotypes and the phenotypic trait values. Moderate levels of trait heritability were estimated between 40 and 74% that correspond with previous studies. Our contemporary naive analysis identified potential additive QTLs on chromosomes *2*, *3*, *5* (top arm) and *9* across the years. Moreover, cofactor QTL analysis identified two masked QTLs on chromosomes *1* and *5* (lower arm). The QTLs on chromosomes *2*, *5* (lower arm) and *9* are reported here for the first time. The QTLs that we identified on chromosomes *1*, *3* and *5* (top arm) show overlap with previous studies for protein content in potato. Collectively the naive QTLs explained 12 to 17% of the phenotypic variance. The underlying alleles of the QTLs provided both positive and negative effects on the phenotype. Our work uncovers the complex genetic architecture of this trait and describes potential breeding strategies for improvement. As protein has emerged as a high-value component from industrial potato starch production, the dissection of the genetic architecture and subsequent improvement of this trait by breeding has great economic and environmental relevance.

**Electronic supplementary material:**

The online version of this article (10.1007/s10681-018-2331-z) contains supplementary material, which is available to authorized users.

## Introduction

Potato (*Solanum tuberosum* L.) is the fourth most important food crop worldwide (FAO 2014) and is becoming increasingly important in developing countries. It is a major source of starch, protein, vitamins and minerals and therefore an important crop for both human consumption and the starch industry (Jørgensen et al. 2006). When starch is industrially extracted from potato tubers, large quantities of potato fruit juice (PFJ) are released as an aqueous by-product that contains soluble protein (Bárta et al. 2012). In the past, PFJ was not valorised by the potato starch industry and was treated as a waste-stream. Consequently, PFJ was discharged into rivers and channels which often resulted in environmental pollution. Nowadays, functional proteins are extracted from PFJ by innovative industrial processors to create added-value in the starch potato production chain. Functional potato proteins are economically valuable as food ingredients due to their techno-functional properties such as gelling behaviour (Creusot et al. 2011), anti-oxidant properties (Kudo et al. 2009) and high nutritional value (Bártová and Bárta 2009). The concentration of soluble protein in PFJ of commercial varieties is known to range from 1 to 1.5% (Ortiz-Medina 2006). Soluble protein in PFJ is generally classified into three main groups consisting of patatin, protease inhibitors and a group of high-molecular weight proteins (Pots et al. 1999). The processing of tubers with high levels of protein content is economically relevant for the potato starch industry as these compounds render high economic value. Therefore, improving protein content in starch potato varieties has emerged as a topic for innovation amongst starch potato breeders. Shedding light on the genetic architecture of protein content—by characterizing underlying QTLs—provides relevant insight for defining strategies on how to improve this trait by means of breeding.

The genetic factors underlying protein content in potato are poorly studied. To the best of our knowledge, only two genetic studies describing QTLs have been published. These studies involved the use of a diploid bi-parental potato mapping population of limited agronomic value (Acharjee et al. 2018; Werij 2011). In diploid populations the broad sense heritability of protein content has been estimated between 56 and 66% (Lu et al. 2012; Werij 2011). These findings indicate that a moderate proportion of the trait variance can be ascribed to genetic factors within a particular experimental setup. Acharjee et al. (2018) and Werij (2011) identified QTLs for protein content on chromosomes *1*, *3* and *5*, illustrating that these genetic loci affect the level of soluble protein content in potato tubers. At present, no QTL study has been reported for protein content in cultivated tetraploid potato. The identification of QTLs in tetraploid potato provides relevant insight for breeding as most crosses are made using tetraploids that contain four sets of homologous chromosomes (2n = 4x = 48). Genetic studies in other crops reveal that protein content is a quantitative trait that is controlled by cumulative actions of both genetic and environmental factors. In soybean, wheat and maize it has been shown that protein content is regulated by multiple loci that are likely to be influenced by genotype-by-environment interactions (Balyan et al. 2013; Hwang et al. 2014; Karn et al. 2017). Marker-assisted breeding for elite varieties with enhanced levels of protein content is therefore challenging without a basic understanding of the genetic architecture and QTLs of the trait.

As potato breeding is almost exclusively performed by making tetraploid crosses, it is relevant to perform genetic studies at a tetraploid level. Performing these studies is challenging due to the complexities of dealing with tetrasomic inheritance, allowing for pairing between all sets of homologous chromosomes, and the computation power needed to analyse vast quantities of marker-data that originate from commonly used single-nucleotide polymorphism (SNP) arrays. In recent years however, great progress has been made in the development of novel, rapid and user-friendly tools for the construction of chromosomal linkage maps from genetic marker-based data and mapping of trait-derived QTLs in outbred tetraploid species that are highly heterozygous (i.e. potato). New tools allow the genetic analysis of large numbers of SNPs available from modern genotyping arrays. These include statistical methods for the construction of high-density SNP-based chromosomal linkage maps (Bourke et al. 2016; Hackett et al. 2013, 2014) and the reconstruction of multi-locus probabilistic haplotypes in outcrossing tetraploids (Zheng et al. 2016). Simulations by Zheng et al. (2016) have illustrated that probabilistic haplotype reconstruction is able to quantify the presence of all possible combinations of parental alleles in the progeny and that it can deal with quadrivalent pairing of four synapsed homologous chromosomes during the first stages of meiosis. This approach is robust in handling the possible but low-frequent occurrence of double reduction products from quadrivalent pairing and in using genetic maps containing some degree of errors and missing allele dosage information in parents and offspring. The application of these methods enables the dissection of the genetic architecture of complex quantitative traits in tetraploid potato.

In this study, we investigated the genetic architecture of protein content by QTL analysis of a large bi-parental tetraploid mapping population (2n = 4x = 48) from a cross between two contrasting commercial varieties. Phenotypic data from 496 F_1_ individuals was collected from field trials that were carried out over 3 consecutive years. Genotypic data originating from a 60K SNP array were transformed into multi-locus probabilistic haplotypes for QTL analysis. This transformation step included SNP dosage scoring, construction of homologue specific linkage maps, construction of an integrated linkage map and estimation of identity-by-descent (IBD). We identified QTLs on several chromosomes and compared these with previous studies. The aim of this study was to shed light on the genetic architecture of protein content in cultivated tetraploid potato by identifying and describing the genetic factors that modulate this trait.

## Materials and methods

### Plant material and field experiments

The complete tetraploid population (2n = 4x = 48) consisting of 972 full-sib F_1_ clones originates from a cross between the varieties Altus and Colomba. The female parent Altus is a starch potato variety (Averis Seeds, Valthermond, The Netherlands). Altus has a high level of tuber protein content and descends from a cross between KA 87-2306 and Kartel. The male parent Colomba is a consumption variety (HZPC, Metslawier, The Netherlands). Colomba has a low level of tuber protein content and results from a cross between Carrera and Agata. Field experiments were conducted in 2012, 2013 and 2014 during the conventional potato growing season in the northern region of the Netherlands (April to September), in Valthe (2012), Nieuw-Weerdinge (2013) and Grolloo (2014). A random selected subset of this population, consisting of 496 F_1_ clones, and the two parental varieties were grown from seed tubers in two randomized blocks in 2013 and 2014 and each consisted of six-hill plots per experimental unit. In 2012 the population was grown in a single block. The trial was well balanced as all but two clones were grown in all 3 years of the trial.

### Quantification of soluble protein content

Soluble protein content was quantified in potato fruit juice (PFJ) using SPRINT™ Rapid Protein Analyser (CEM Corporation, NC, USA). Purified potato tuber protein (AVEBE, Veendam, The Netherlands) was used as a standard. Each PFJ sample was measured in two technical replicates. PFJ samples were extracted from 5 kg batches of representative fresh tubers from all individual F_1_ clones and both parental varieties after measuring the fresh weight and under-water weight.

The tubers were sliced, mixed and processed with a juice extractor (Rotor Lips Ltd., Uetendorf, Switzerland) and PFJ was collected directly. The tubes containing the PFJ samples were kept cold on ice. After 10 min of settling time, a second PFJ sample was collected from the supernatant phase of the sample and 1% (v/v) of 5% sodium metabisulfite (w/v) was added to inhibit enzymatic browning. The PFJ was then centrifuged at 15,000×*g* for 5 min and the supernatant was collected and stored at − 20 °C until use. Tuber dry matter was inferred from tuber under-water weight as described in previous studies (Bradshaw et al. 2008; Sverrisdóttir et al. 2017). The tuber moisture content was computed as follows:

$$Tuber\;moisture\;content = 100 - Tuber\;dry\;matter$$

where tuber moisture content and tuber dry matter are expressed in percentages.

The phenotypic values of protein were computed by correcting protein content in PFJ by tuber moisture content as follows:

$$Protein\;content = \frac{Protein\;content\;in\;PFJ \times Tuber\;moisture\;content}{100}$$

where protein content in PFJ is expressed in milligram protein per milliliter PFJ (1% = 10 mg/ml PFJ) (w/v) and tuber moisture content in percentages.

### Variance components and trait heritability

The variance components of protein content were computed from the mean squares (MS) values as output from the one-way (within year) and two-way analysis (between years) of variance (ANOVA). The variance components were computed as follows: *σ*_*E*_^2^ = MS_E_, $\sigma_{{G\;{\text{one - way}}}}^{2} = \frac{{MS_{G} - MS_{E} }}{r}$, $\sigma_{{G\;{\text{two}}\;{\text{way}}}}^{2} = \frac{{MS_{G} - MS_{G*Y} }}{r*y}$, $\sigma_{{G*Y\;{\text{two}}\;{\text{way}}}}^{2} = \frac{{MS_{G*Y} - MS_{E} }}{r}$, where *σ*_*G*_^2^, *σ*_G*Y_^2^ and *σ*_*E*_^2^ are the variance components of the F_1_ clones, F_1_ clones by year interaction and residuals respectively. The number of years and the number of biological replicates are expressed in the terms *y* and *r* respectively. The following ANOVA models were used for analysis:

$$\begin{aligned} ANOVA\;within\;year :\;y_{ij} & = \mu + \tau_{i} + \alpha_{j} + \varepsilon_{ij} \\ ANOVA\;between\;years :\;y_{ijk} & = \mu + \tau_{i} + \beta_{k} + \alpha \beta_{jk} + \tau \beta_{ik} + \varepsilon_{ijk} \\ \end{aligned}$$

where *y*_*ij*_ and *y*_*ijk*_ represent the protein content values of the *i*-th clone, in the *j*-th block, in the *k*-th year, *µ* is the overall mean response, *τ*_*i*_ is the effect of the *i*-th clone, *α*_*j*_ is the effect of the *j*-th block, *β*_*k*_ is the effect of the *k*-th year, *αβ*_*jk*_ is the nested effect of the *j*-th block in the *k*-th year, *τβ*_*ik*_ is the effect of the interaction between the *i*-th clone of the *k*-th year and *ε*_*ijk*_ is the random error term. The broad sense heritability estimates (*H*^2^) were computed as follows:

$$H^{2} \;within\;year = \frac{{\sigma^{2}_{G} }}{{\left( {\sigma^{2}_{G} + \frac{{\sigma^{2}_{E} }}{r}} \right)}},$$

$$H^{2} \;between\;years = \frac{{\sigma^{2}_{G} }}{{\left( {\sigma^{2}_{G} + \frac{{\sigma^{2}_{G*Y} }}{y} + \frac{{\sigma^{2}_{E} }}{r*y}} \right)}} .$$

The phenotypic values of protein content were collected from multi-year field trials at the three different locations. A mixed model was used to obtain best linear unbiased estimates (BLUEs) with adjusted mean values for the F_1_ clones for the phenotypic values from 2012 to 2014. The estimation was done using restricted maximum likelihood (REML) to compute the response as reported (Björn et al. 2011):

$$Response = Genotype + Year + Error$$

where the genotype effect is fixed and year effects and errors are random terms.

### Genotyping using a single-nucleotide polymorphism (SNP) array

Genotyping of the complete population of 972 individuals and the parental varieties was performed with the 60K Axiom SNP marker array. This array consists of a subset of the 20K SNPs from the SolSTW Infinium SNP array (Vos et al. 2015) and an additional 40K SNPs that originated from RNA sequences of both parental varieties used in this study (unpublished data).

### Data processing and genotype calling

Allele dosage scores were assigned to the SNP markers using the fitTetra R package (Voorrips et al. 2011) as previously described (Bourke et al. 2015). SNP allele dosage scores assigned by fitTetra were tested with the function CheckF1 (Bourke et al. 2018b) in R to identify the best-fitting segregation model for the SNPs. SNPs that did not correspond to the assumed segregation were discarded. SNPs with high skewness (using a Chi square test, *α* = 0.001) or more than 5% missing values were removed. Also, F_1_ clones with more than 10% missing SNPs were removed. The complete mapping population of 972 individuals was used to construct a high-density tetraploid integrated chromosomal linkage map.

### Chromosomal linkage maps and identity-by-descent estimation

The complete population of 972 F_1_ clones was used for constructing the chromosomal linkage maps according to methods described by Bourke et al. (2016) with minor modifications. First, simplex × nulliplex markers were assigned to 12 putative chromosomal clusters at a linkage LOD score threshold of 10, after which they were separated into putative homologue clusters at a LOD score threshold of 30. Per chromosome, pairwise recombination frequencies and LOD scores were calculated between all marker segregation types and marker alleles were phased and assigned to homologues. Next, a developmental version of the MDSmap software (Preedy and Hackett 2016) was used to order the markers. This resulted in twelve integrated chromosomal linkage groups representing the twelve potato chromosomes. These maps were produced using unconstrained weighted metric multi-dimensional scaling with the squared LOD scores for linkage as weights and using Haldane’s mapping function. This was followed up by principal curve fitting in two dimensions to order the markers. Outlying markers in principal curve analysis, as judged by the eye, and those with a nearest-neighbour fit exceeding 5 were removed to select only high-quality markers as described by Preedy and Hackett (2016). Up to three rounds of MDSmap were performed until all outlying markers were removed. After the first and second round of MDSmap, in total 86 and 30 markers, respectively, were removed as possible outliers. In the third round, no further outliers were identified, resulting in stable integrated chromosomal linkage maps. Linkage groups were renumbered according to the reference potato genome sequence (PGSC 2011) using the known assignments of SNPs on the physical map containing the DNA sequence assembly of the twelve potato chromosomes (PGSC pseudomolecules v4.03).

The identity-by-descent (IBD) probabilistic haplotypes were estimated using TetraOrigin (Zheng et al. 2016). SNPs from each possible segregation type were selected at centiMorgan (cM) map positions (rounded off to 1 decimal place), with preference given to markers with the smallest amount of missing data whenever multiple markers occupied the same position. TetraOrigin (Zheng et al. 2016) was run in Mathematica version 10 (Wolfram Research Inc., Champaign, Illinois, USA) with bivalentPhasing set to True and bivalentDecoding set to False for taking into account the occurrence of double reduction in the probabilistic haplotypes of the F_1_ clones. The allele dosage error probability for both parents (*epsF*) and F_1_ clones (*eps*) were set to 0 and 0.001 respectively (Bourke 2018). These setting were used due to the high quality (confidence) dosage scores that were assigned to both parents from technical replicates (*N *= 12) of the SNP array. Moreover, these setting have been shown to be effective and appropriate for use in TetraOrigin as demonstrated by Zheng et al. (2016). For the other parameters, the default settings were used (*maxStuck* = 10, *maxIteration* = 100, *minRepeatRun* = 3, *maxPhasingRun* = 20).

### Naive QTL analysis

A naive single-locus QTL analysis was carried out for all chromosomes. This analysis was carried out using the IBD probabilistic haplotypes produced by TetraOrigin, after splines were fitted on a grid at cM map positions. The model used has previously been described as Kempthorne’s “additive model” (Hackett et al. 2013, 2014), with the difference that all possible genotypes under a quadrivalent model allowing for double reduction were included. The terms in the model correspond to the haplotype probabilities *X*_*i*_ from Altus (parent 1: 1 ≤ *i* ≤ 4) and Colomba (parent 2: 5 ≤ *i* ≤ 8); we relate the phenotypes of the F_1_ clones to the haplotype probabilities in the following manner (Single-locus QTL model A):

$$\begin{aligned} & Single - locus\;QTL\;model\;A :\\ & \quad y = \mu + \alpha_{1} X_{1} + \alpha_{2} X_{2} + \alpha_{3} X_{3} + \alpha_{4} X_{4} + \alpha_{5} X_{5} + \alpha_{6} X_{6} + \alpha_{7} X_{7} + \alpha_{8} X_{8} + \varepsilon \\ \end{aligned}$$

Given the constraints $\sum\nolimits_{i = 1}^{4} {X_{i} = 2}$ and $\sum\nolimits_{i = 5}^{8} {X_{i} = 2}$, two terms were eliminated to avoid over-parametrization and co-linearity for regaining independence between the explanatory variables (Single-locus QTL model B), so that:

$$\begin{aligned} & Single - locus\;QTL\;model\;B :\\ & \quad y = \mu^{\prime } + \alpha_{2}^{\prime } X_{2} + \alpha_{3}^{\prime } X_{3} + \alpha_{4}^{\prime } X_{4} + \alpha_{6}^{\prime } X_{6} + \alpha_{7}^{\prime } X_{7} + \alpha_{8}^{\prime } X_{8} + \varepsilon . \\ \end{aligned}$$

For the QTL analysis a genome-wide significance threshold was determined by permutation testing on the phenotypic values with *N* = 1000 cycles and *α *= 0.05 (Churchill and Doerge 1994). The QTL analysis was performed over a sliding window at 1-unit cM intervals. The minimum Schwarz information criterion (Schwarz 1978), also known as the Bayesian information criterion (BIC), was used to explore the most likely to be bi-allelic QTL model (of all possible combinations) and the origin of its effect—both additive and dominant—in a similar manner as described by Hackett et al. (2014).

The phenotypic difference between the average homologue effect ($\bar{h}$) on the overall mean trait value of the population ($\bar{y}$) was estimated simultaneously for all homologues of chromosomes containing a QTL as: $\bar{h} - \bar{y}$, by using the following formula:

$$\bar{h} = \frac{{\mathop \sum \nolimits_{i = 1}^{N} \pi_{i} y_{i} }}{{\mathop \sum \nolimits_{i = 1}^{N} \pi_{i} }}.$$

The terms in the model correspond to the IBD probabilities ($\pi_{i}$), the trait values of the F_1_ clones ($y$) and the homologues from Altus (parent 1: 1 ≤ *i* ≤ 4) and Colomba (parent 2: 5 ≤ *i *≤ 8). The obtained results were plotted to visualize the effects of the homologues. The genotypic information coefficient (GIC) was computed (Bourke et al. 2018a) to provide insight into the information density to infer QTL effects across the mapped genome.

### Cofactor QTL analysis

A cofactor QTL analysis that allows for multiple QTLs as cofactors (Jansen and Stam 1994) was carried out in a stepwise procedure. First a naive single-locus QTL analysis (model B) was performed on the protein content values of BLUEs 2012–2014. The positions of significant QTL peaks derived from the naive QTL analysis were defined as cofactors in a subsequent analysis. The residuals resulting from this analysis were saved. Subsequently these residuals were analysed once again using the naive single-locus QTL analysis model B. A genome-wide significance threshold was determined by permutation testing on these residuals with *N* = 1000 cycles and *alpha* = 0.05 (Churchill and Doerge 1994).

### Computation of LOD scores

The LOD scores for all the QTL analyses were computed (Broman et al. 2003), so that:

$$LOD = \frac{n}{2}log_{10} \left( {\frac{{RSS_{0\;model} }}{{RSS_{1\;model} }}} \right).$$

where $RSS_{0\;model}$ is the residual sum of squares for the null model (no QTL) and $RSS_{1\;model}$ is the residual sum of squares from the fit of the full model.

## Results

### Integrated linkage map construction

**Fig. 1:**
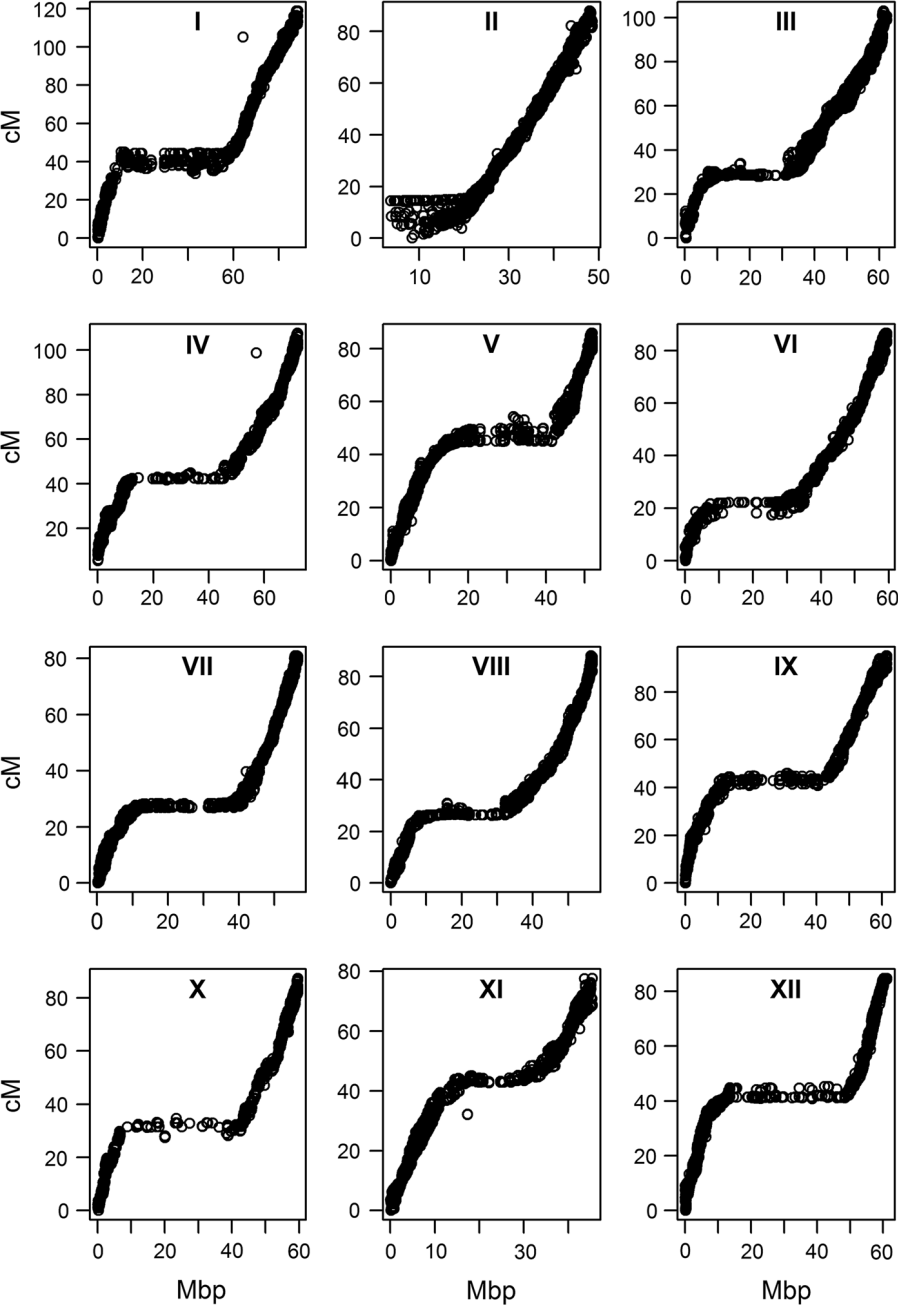
Plots of the genetic location (cM) versus physical position (Mbp) of SNPs across the chromosomes. The twelve chromosomes of potato are shown in the boxes. The horizontal stretches in the plotted data represent the centromeric regions on the chromosomes

The integrated linkage map (Table 1) consisted of 23,328 high quality segregating SNPs. For Altus the parental-specific SNPs included simplex × nulliplex and duplex × nulliplex and for Colomba these SNPs included nulliplex × simplex and nulliplex × duplex. Herewith Altus contributed 10,360 SNPs and Colomba 7998 SNPs. The mapped SNPs were not distributed equally over the chromosomes and homologues of each chromosome. The number of SNP markers ranged between 1204 and 2916 between the chromosomes. Also the density of SNPs across homologues of the same chromosomes were variable (Table 1). The genetic location (cM) and physical position (Mbp) of the SNPs mapped as expected and contained only a few outliers that deviated from the expected patterns from the genetic versus physical positions of the markers (Fig. 1). The centromeric regions of the potato chromosomes (Sharma et al. 2013)—characterized by the absence of recombination—could be clearly identified as visible horizontal stretches in the figure.

**Table 1:** Linkage map summary of the parental varieties Altus and Colomba

Chr.	Total^a^	Altus^b^	Colomba^b^	Altus^c^				Colomba^c^				Length of integrated chr. linkage map (cM)	Length of physical chr. map of PGSC v4.03 (Mb)
				h1	h2	h3	h4	h1	h2	h3	h4		
1	2780	981	982	384	519	213	232	344	285	179	397	118.8	88.7
2	2604	1410	705	331	650	556	100	237	325	237	231	88.0	48.6
3	2916	1706	775	389	788	254	239	181	216	118	331	103.0	62.3
4	1985	905	631	121	293	228	344	200	61	391	200	107.5	72.2
5	1743	751	705	288	257	173	115	164	208	301	77	85.8	52.1
6	1991	1059	538	365	458	200	179	228	144	130	148	86.6	59.5
7	1814	807	600	310	192	209	117	123	167	247	227	81.0	56.8
8	1675	616	723	231	100	222	214	190	103	337	207	88.1	56.9
9	1778	733	639	335	275	165	94	183	156	317	124	95.2	61.5
10	1204	355	499	197	77	126	81	183	146	124	158	87.3	59.8
11	1454	574	608	217	130	119	143	171	278	98	151	77.6	45.5
12	1384	463	593	115	80	194	209	187	117	185	103	84.8	61.2
Total	23,328	10,360	7998	3283	3819	2659	2067	2391	2206	2993	2354	1103.7	725.1

Table S1 provides detailed information of the phased SNP markers. Chr. = chromosome. h1 to h4 = homologue 1 to homologue 4

^a^Number of mapped markers on the integrated chromosomal linkage maps

^b^Markers with segregating alleles from one parent only which include simplex × nulliplex and duplex × nulliplex in the parent under consideration

^c^Number of homologue-specific markers in simplex condition in the parent under consideration which includes alleles from simplex × nulliplex, simplex × simplex, simplex × duplex and simplex × triplex in one parent and vice versa for the other parent

### Phenotypic values of protein content

**Fig. 2:**
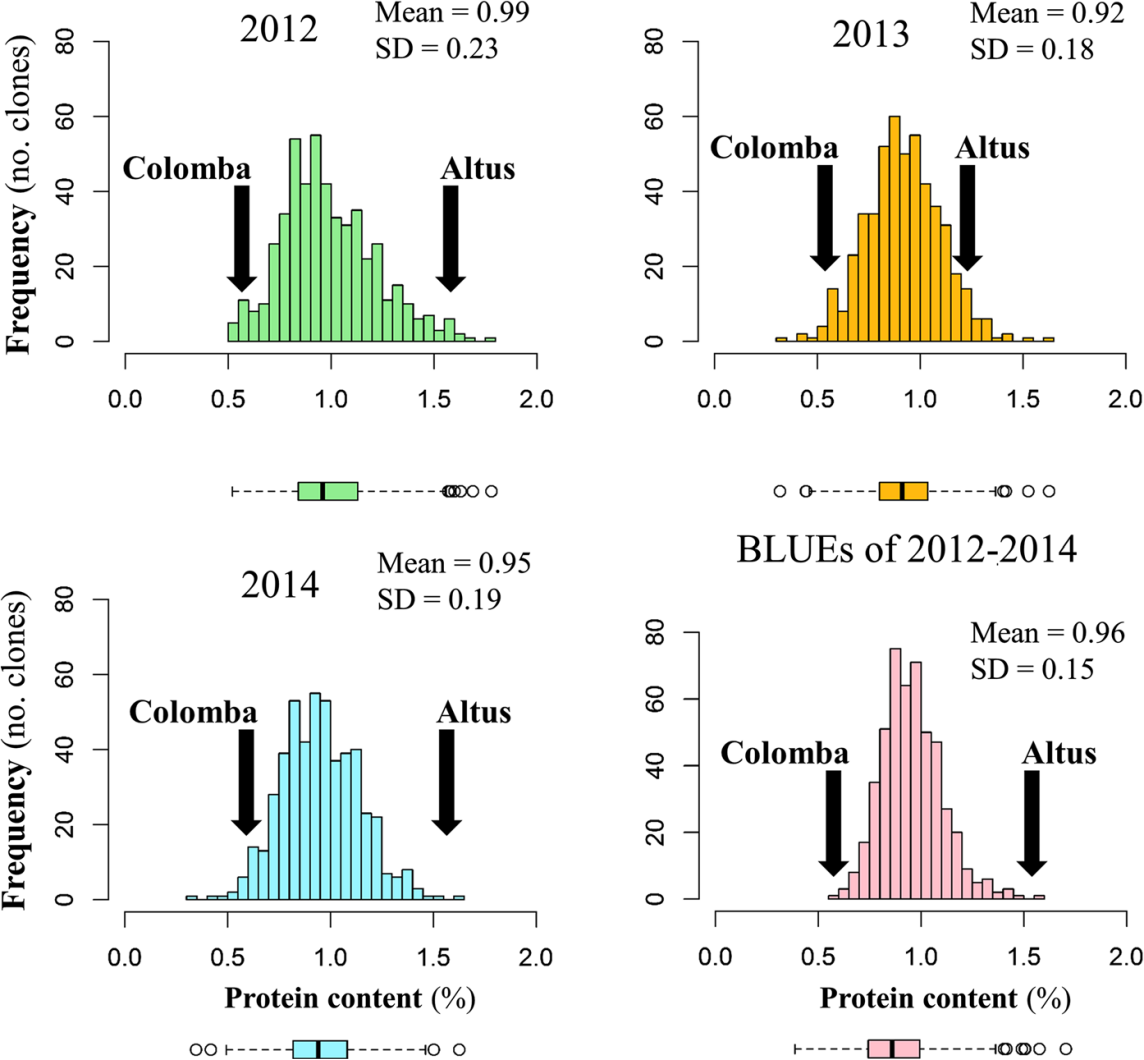
Distributions of protein content across the years. The green colour stands for year 2012, the orange colour stands for year 2013, the blue colour stands for year 2014 and the pink colour stands for the BLUEs of the years 2012 to 2014. Corresponding box plots are shown below the histogram figures. The arrows indicate the mean values for the parental varieties Altus (parent 1) and Colomba (parent 2). *SD* standard deviation. (Color figure online)

We did not observe spatial trends for the phenotypic values in the field trials. The distribution of the trait valued did not show any (clear) segregation patterns and followed approximately a normal distribution (Fig. 2). Compared to the parental varieties, extreme high and low trait values of the F_1_ clones were observed in both 2012 and 2013. In contrast, only extreme low trait values were found in 2014.

Significant effects of the clones were both within (*P* = 5 × 10^−7^ for 2013 and 2014) and between (*P* = 5 × 10^−7^ for 2013–2014) the years (Table 2). The broad sense heritability estimate was 40% for 2013, 55% for 2014 and 74% over 2013 and 2014. Evidence for clone-by-year interaction was not found (*P *= 0.395 for 2013–2014). The data for 2012 was not included for heritability estimation as the population lacked replication in the field for this year (*N *= 1).

**Table 2:** Summary statistics of protein content for the years 2013, 2014 and 2013–2014

Parameter	2013	2014	2013–2014
Min.	0.32	0.35	0.58
Mean	0.92	0.95	0.93
Max.	1.63	1.63	1.63
σ_G_^2^	0.013***	0.021***	0.042***
σ_GxY_^2^	–	–	0.001 N.S.
σ_ε_^2^	0.039	0.034	0.062
*H* ^2^	0.40	0.55	0.74

σ_G_^2^ = Genotype/clone variance, σ_GxY_^2^ = Genotype/clone by Year interaction variance, σ_ε_^2^ = Residual variance, *H2* = Broad sense heritability estimate

***P < 0.001, *N.S.* non-significant: *P *> 0.05

**Fig. 3:**
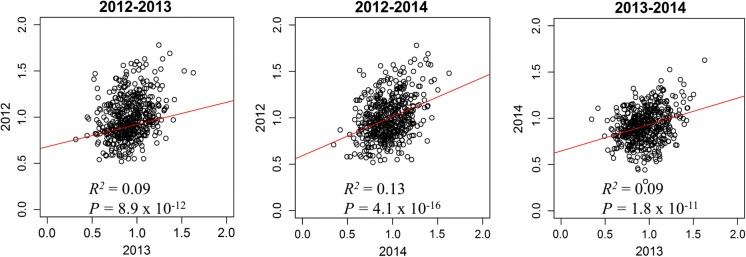
Plots of the values of protein content between the years 2012–2013, 2012–2014 and 2013–2014. The red line represents the simple linear regression line. (Color figure online)

Correlation analyses of phenotypic values between the years revealed low to poor *R*^2^ values that significantly deviated from zero (2012–2013: *R*^2^ = 0.09, *P *= 8.9 × 10^−12^; 2012–2014: *R*^2^ = 0.13, *P *= 4.1 × 10^−16^; 2013–2014: *R*^2^ = 0.09, *P *= 1.8 × 10^−11^) (Fig. 3).

### Naive QTL analysis and variance explained by QTLs

**Fig. 4:**
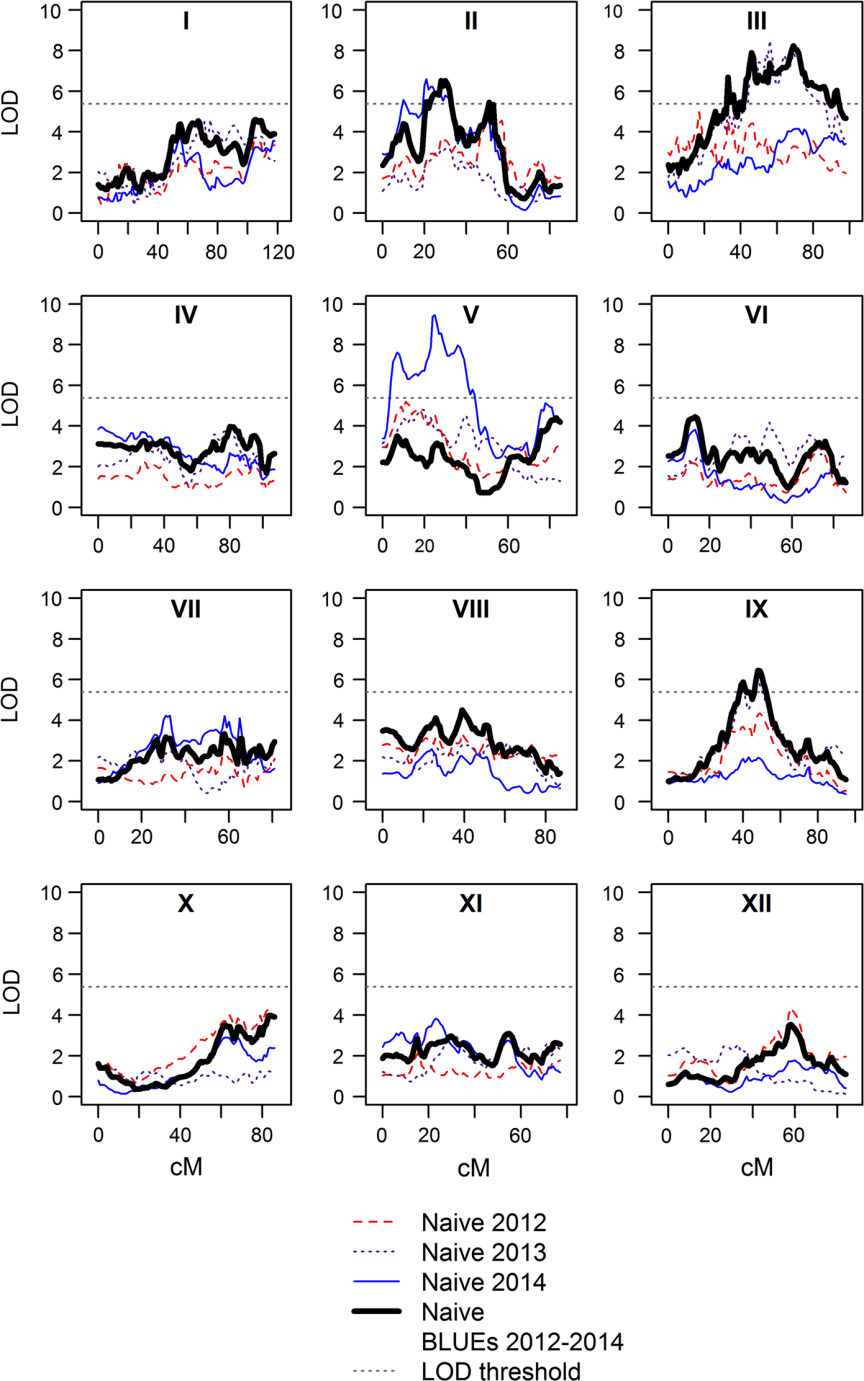
LOD profiles of the naive single-locus QTL analysis. The boxes represent the twelve individual chromosomes of potato. The red dashed line represents the year 2012, the purple dotted line represents the year 2013, the blue line represents the year 2014 and the black line in bold represents the BLUEs of the years 2012–2014. The horizontal dashed line represents the permutation-based LOD threshold. (Color figure online)

Naive QTL analysis was performed by regression analysis (single-locus QTL model B)—by using the phenotypic values and the IBD probabilistic haplotypes—at all positions on the chromosomes. Separate analyses were conducted for protein content in 2012, 2013, 2014 and the BLUEs of 2012–2014. QTLs were detected on chromosomes *2*, *3*, *5* and *9* (Fig. 4) for 2013, 2014 and the BLUEs of 2012–2014 (Table 3). No significant QTLs were found for 2012. The largest QTL was found on chromosome *5* for 2014, however in the other years this QTL was not found.

**Table 3:** Statistics of QTLs from the naive single-locus QTL analysis

Year	Chr.	LOD peak position and LOD-2 interval (cM)	LOD score	*R* ^2^
2013	*3*	56: 44–75	8.4	0.076
2013	*9*	48: 47–53	5.9	0.051
2014	*2*	21: 9–33	6.6	0.059
2014	*5*	25: 22–39	9.5	0.084
BLUEs 2012–2014	*2*	30: 19–54	6.5	0.058
BLUEs 2012–2014	*3*	69: 32–82	8.2	0.074
BLUEs 2012–2014	*9*	48: 34–55	6.5	0.058

Naive represents naive QTL analysis without cofactors. Chr. represents chromosome. LOD represents logarithm of the odds. LOD-2 interval *spans the* support interval above the QTL threshold. BLUEs represent best linear unbiased estimates

**Fig. 5:**
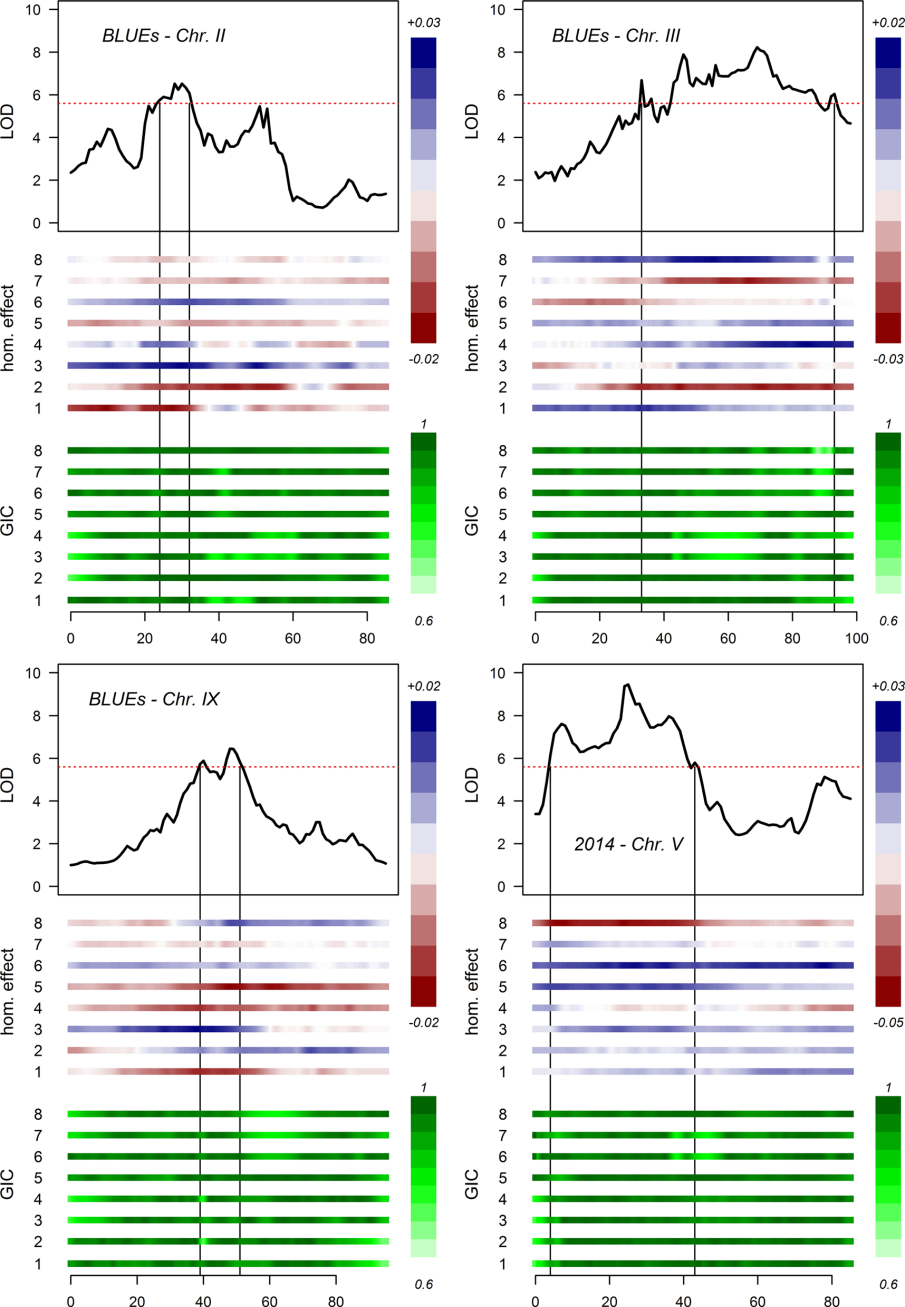
Contribution of homologues on the values of protein content for the QTLs identified on chromosomes *2* (top left), *3* (top right), *5* (bottom right) and *9* (bottom left). Homologues (hom.) 1 to 4 originate from Altus (parent 1) and homologues 5 to 8 from Colomba (parent 2). The upper vertical scale in blue to red represents the contribution of the homologues on the values of protein content. The lower vertical scale in dark green to light green represents the genotypic information coefficient (GIC) of the homologues. The horizontal red dashed line in the LOD plot represents the permutation-based LOD threshold. The horizontal scale below the homologue box represents the genetic (cM) positions. (Color figure online)

Numerous underlying haplotypes (or alleles) of the QTLs found on chromosomes *2*, *3*, *5* and *9* provided positive or negative effects on the trait values (Fig. 5). For the strongest QTL (chromosome *5* from 2014), the haplotypes originating from Colomba (parent 2) on homologues *5* and *6* provided positive effects, whilst homologue 8 provided a negative effect.

The percentage of phenotypic variation (*R*^2^) explained by individual QTLs ranged between 5.1 and 8.4% (Table 3). The largest amount of variation (8.4%) was explained by the QTL on the top arm of chromosome *5* at 25 cM in 2014. The QTLs on chromosome *3* explained 7.6% and 7.4% in 2013 (56 cM) and BLUEs of 2012–2014 (69 cM) respectively. The QTLs on chromosomes *2* and *9* explained between 5.1 and 5.9% of the phenotypic variation. The most probable QTL segregation models of the QTLs in Table 3 were assessed by means of the minimum Schwarz information criterion (SIC) to assess the strength of evidence of the effects and origins of the parental haplotypes that contribute towards the trait. The differences in the minimum SIC of segregation models with balanced group sizes revealed values that are considered low (less than 2—data not shown) (Neath and Cavanaugh 2012) and are therefore not elaborated on. However, one exception was found for chromosome *5* in 2014 with a segregating QTL—QQQQ × QQQq—Colomba (parent 2). This model provided a minimum SIC value of 19 that is considered as a probable segregation model for this QTL. This segregation model accounted for a mean protein content value of 0.905 for ‘q’ versus 0.995 for ‘Q’ of the segregating allele on homologue four from Colomba. This finding is consistent with the negative effect of homologue *8* for this QTL in 2014 from Colomba (Fig. 5).

The variation explained by all QTLs for 2013 (chromosomes *2* and *9*), 2014 (chromosomes *2* and *5*) and BLUEs of 2012–2014 (chromosomes *2*, *3* and *9*) accounted for 11.9%, 12.7% and 17.2% of the total phenotypic trait variation respectively.

### QTL cofactor analysis

**Fig. 6:**
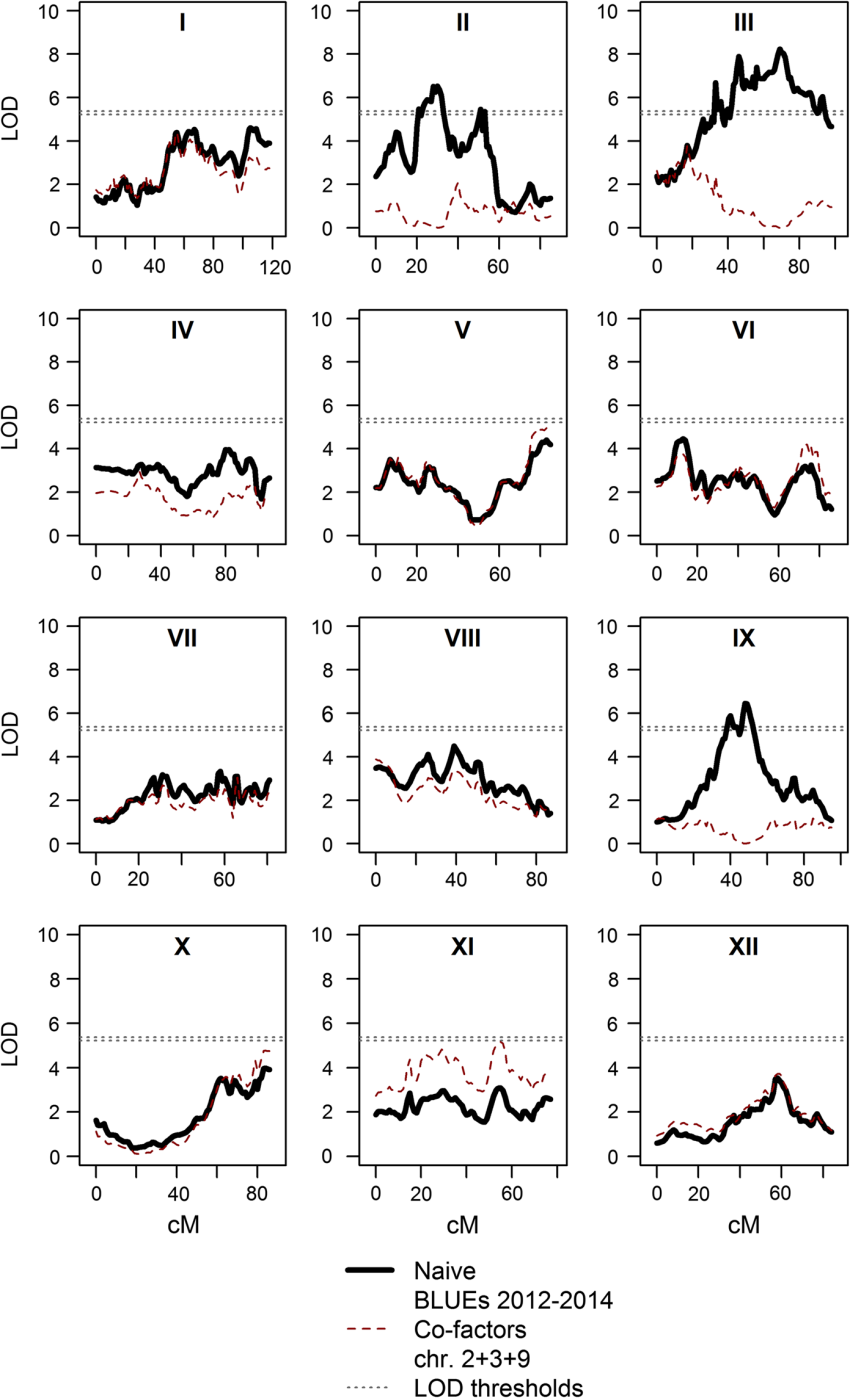
LOD profiles of both the naive and cofactor QTL analysis for the BLUEs of the years 2012–2014. The black line in bold represents the LOD scores of the naive QTL analysis and the red dashed line represents the LOD scores of the cofactor QTL analysis with three peak QTLs set as cofactors from chromosomes *2*, *3* and *9* (Table 3). The top horizontal dashed line shows the permutation-based LOD threshold for the naive QTL analysis and the lower horizontal dashed line shows the permutation-based LOD threshold for the cofactor QTL analysis. (Color figure online)

To identify masked QTLs, cofactor analysis was performed on the protein content values for 2013, 2014 and the BLUEs of 2012–2014 by using the QTLs identified by the naive analysis (Table 3) as cofactors (Jansen and Stam 1994). No masked QTLs were revealed by cofactor analysis of the BLUEs 2012–2014 when the QTLs on chromosomes *2*, *3* and *9* were set as cofactors (Fig. 6). However, two masked QTLs were identified on chromosomes *1* (Fig. S4) and *5* (Fig. S11) after other combinations of QTLs were used as cofactors (Table 4).

**Table 4:** Statistics of QTLs from cofactor analysis

Year	Naive QTL(s) as cofactor(s): chr. (position)^a^	LOD scores of QTLs by cofactor analysis (chr. and position)^b^	*R*^2^ of QTLs by cofactor analysis^c^	New QTLs identified by cofactor analysis: chr. (position)^d^
2013	*3* (56)	5.7 (*9*-40) Fig. S1	0.051	No
2013	*9* (48)	7.8 (*3*-56) Fig. S2	0.07	No
2013	*3* (56) + *9* (48)	N.S. Fig. S3	N.S.	No
2014	*2* (21)	9.3 (*5*-24); 5.5 (*5*-78) Fig. S4	0.083; 0.050	*5* (78)
2014	*5* (25)	6.2 (*2*-23) Fig. S5	0.056	No
2014	*2* (21) + *5* (25)	N.S. Fig. S6	N.S.	No
BLUEs 2012–2014	*2* (30)	5.4 (*1*-67); 9.1 (*3*-69); 6.3 (*9*-40) Fig. S7	0.049; 0.081; 0.056	*1* (67)
	*3* (69)	6.8 (*2*-30); 5.7 (*9*-40) Fig. S8	0.062; 0.052	No
	*9* (48)	6.2 (*2*-30); 7.4 (*3*-69) Fig. S9	0.056; 0.067	No
	*2* (30) + *3* (69)	6.1 (*9*-40) Fig. S10	0.059	No
	*2* (30) + *9* (48)	8.2 (*3*-69); 5.9 (*5*-83) Fig. S11	0.074; 0.053	*5* (83)
	*3* (69) + *9* (48)	6.7 (*2*-28) Fig. S12	0.06	No
	*2* (30) + *3* (69) + *9* (48)	N.S. Fig. 6	N.S.	No

Chr., chromosome; Position, QTL peak position in centiMorgan (cM); LOD, logarithm of the odds; BLUEs, best linear unbiased estimates; N.S., non-significant

^a^QTL(s) identified by naive QTL analysis (Table 3)

^b^QTL(s) identified by cofactor QTL analysis after one or more QTL(s) identified by naive QTL analysis were used as cofactors

^c^Variance explained (*R*^2^) of QTL(s) identified by cofactor QTL analysis

^d^New QTLs identified by cofactor QTL analysis that were not identified by naive QTL analysis

## Discussion

The development of elite potato varieties with high levels of protein content is an innovative topic of great economic and environmental relevance for the potato starch industry. Hence protein content in potato has emerged as a novel breeding goal. Shedding light on the genetic architecture underlying this trait and identifying QTLs are the first necessary steps for defining strategies that may enable (marker-assisted) breeding of elite varieties with high protein content. In this study, we evaluated the genetics of protein content for a large bi-parental mapping population of 496 full-sib F_1_ clones. These clones originate from a cross between two genetically divergent cultivated tetraploid potato varieties with a high and low level of protein content. An integrated chromosomal linkage map was constructed and was used to compute probabilistic haplotypes across all chromosomes that were used for QTL analysis. Potential QTLs were found on five chromosomes and the formation of extreme trait values—i.e. transgressive segregation—was observed in all three years. The occurrence of the extreme trait values in the progeny may be caused by transgressive segregation due to complementary action of additive alleles contributed by different parental varieties. We report broad sense heritability estimates of 40% (2013) and 55% (2014) and 74% (2013–2014), indicating that a moderate proportion of the trait variance can be ascribed to genetic factors. Therefore it can be postulated that breeding for this complex quantitative trait is theoretically possible.

### Chromosomal linkage map and probabilistic haplotypes

The 60K SNP marker array provided a wealth of information for the construction of the integrated chromosomal linkage map that was subsequently transformed into multi-locus probabilistic haplotypes. Only well-performing polymorphic SNPs were used for genotype calling after the removal of monomorphic SNPs and those with too many missing values. This relatively strict procedure resulted in a smaller sub-set of high-quality SNPs—23,328 in total—that were used for linkage mapping and subsequent QTL analysis. Preferably, a smaller set of high-quality SNPs is to be used for linkage mapping instead of a larger set containing potentially erroneous markers (Preedy and Hackett 2016). This was observed in the process of marker ordering for the linkage maps. After three rounds of MDSmap, merely 0.5% (116 SNPs) from the total sub-set of 23,328 SNPs were removed as possible outliers. Our final integrated chromosomal linkage map corresponds well to the physical reference map of potato (PGSC pseudomolecules v4.03). No structural differences—such as noticeable translocations, inversions, insertions or deletions—between our map and the reference genome were observed. The total genetic distance of the map here (1104 cM) is comparable to the length reported in genetic maps of tetraploid potato (1042 to 1088 cM) using fewer SNPs and populations of smaller sizes (Hackett et al. 2013; Massa et al. 2015; Rak et al. 2017). To the best of our knowledge, we present here the most marker-dense potato genetic linkage map and includes SNPs of all possible allele dosage types that segregate from both parents.

TetraOrigin (Zheng et al. 2016) was used to estimate multi-locus probabilistic haplotypes across all chromosomes with high levels of genotype information content across all chromosomes (data not shown). The application of probabilistic haplotypes in genetic association studies in diploids allows for higher statistical power than single-marker procedures, as has been shown in human studies (de Bakker et al. 2005). In tetraploids this application is also expected to generate an equal or higher level of true statistical power than single-marker procedures as information of all 8 homologues is used (Bourke 2014).

To avoid the identification of false-positive QTLs, a stringent genome-wide LOD threshold was estimated from 1000 permutation cycles. The application of this stringent threshold for QTL detection may result in less power to detect minor QTLs, possibly leading to a higher type II error rate (i.e. false negatives). However, results from our co-factor QTL analysis did not reveal any other masked QTLs when compared to the results derived from the naive QTL analysis. The high marker-density of the integrated chromosomal genetic map and final definition of the multi-locus probabilistic haplotypes, in combination with a large mapping population, provides a framework for conducting a reliable QTL analyses with great power. This presumption was found to be true after QTL peaks were mapped only 120 kb away from the well-known potato maturity locus (Cycling DNA-binding with one finger Factor: CDF) at the top of chromosome *5* (data not shown).

### Size of the mapping population

Genetic studies on protein content in other crops such as soybean, wheat and maize illustrate that this trait is quantitative and that it is regulated by multiple genes that are likely to be influenced by genotype-by-environment interactions (Balyan et al. 2013; Hwang et al. 2014; Karn et al. 2017). Therefore, the use of a relatively large mapping population to study this quantitative trait has strongly contributed towards detecting potential QTLs. Moreover, QTL effects are expected to be estimated more accurately in this large population. The use of smaller populations may cause false inflation of QTL effects (Hackett et al. 2014; Vales et al. 2005). The presence of potential epistatic interactions of the QTLs was not analysed in this study. Knowledge on epistasis is relevant to assess whether and which combinations of haplotypes contribute positively or negatively towards the trait. The availability of novel statistical frameworks and computation power are needed to answer these questions for tetraploid potato.

### QTLs for protein content

The factors underlying the genetic architecture of protein content in potato are poorly understood. This study provides insight into the first QTLs for protein content in cultivated tetraploid potato. By using a large bi-parental (Altus × Colomba) mapping population consisting of 496 tetraploid F_1_ clones, we detected potential naive QTLs on chromosomes *2*, *3*, *5* and *9*, each explaining between 5.1 and 8.4% of the trait variance. The variance explained by these QTLs together (11.9 to 17.2%) does not reflect the trait heritability estimates (40 to 74%) that express the amount of variance that can be ascribed to genetic factors. This gap may be caused by factors that include the lack of power to detect minor effect QTLs (by naive QTL analysis), epistatic interactions and genotype-by-environment interactions. QTL cofactor analysis in tetraploid potato may enable the identification of masked QTL for complex traits such as protein content as demonstrated here by cofactor QTL analysis that identified masked QTLs on chromosomes *1* (Fig. S7) and *5* on the lower arm (Fig. S4; Fig. S11). Another explanation for this gap may lie in the heritability estimates themselves. These estimates may be overestimated. Moreover, they do not discriminate between the parts of the trait variance that are heritable and those that are environmental. In the case of overestimation, thus leading toward phantom heritability, the variance explained solely by QTLs may reflect the biology of the trait more accurately.

Previous QTL studies on protein content in non-cultivated diploid potato (Acharjee et al. 2018; Werij 2011) reported QTLs on chromosomes *1*, *3* and *5* (top arm). Whether the haplotypes that account for the QTLs on chromosomes *1*, *3* and *5* in this study are identical to those found in diploid studies requires further research. The QTLs detected in this study on chromosomes *2*, *5* (lower arm) and *9* are novel as they have not been reported in literature before and are presented here for the first time.

The strongest QTL here was found in 2014, a year characterized by ample precipitation at the start of the potato growing season. This QTL was detected at the top arm of chromosome *5* that harbours the major regulator of maturity and initiation of tuber formation (*StCDF1*) as well as a cluster of nitrate transporter genes. In potato, *StCDF1* has been shown to cause pleiotropic effects on multiple sub-traits, including foliar senescence, plant cycle length, the onset of tuberization and potato tuber yield (Hurtado et al. 2012; Kloosterman et al. 2013). In soybean, it has been demonstrated that the plant cycle length and ambient temperature affects protein content during seed development (Patil et al. 2017). In case of *StCDF1*, this QTL may have caused developmental instability that overshadowed other potential QTLs that were found in other years (e.g. QTLs on chromosomes *3* and *9*). Nitrate transporters may also influence the level of protein content. In rice it has been demonstrated that over-expression of a nitrate transporter increased the yield and nitrogen-use efficiency by 40% (Fan et al. 2016). The QTL on chromosome *3* overlaps with regions that harbour gene clusters of tuber proteins that include Kunitz-type protease inhibitors, potato protease inhibitor I (PI-1) and potato protease inhibitor II (PI-2). Protease inhibitors have been suggested to function as storage proteins (Pusztai 1972) as well as potential regulators of proteolysis by means of their inhibition activity against proteases (e.g. trypsin, α-chymotrypsin and elastase). The QTL on chromosome *9* also co-localizes with gene clusters of potato PI-1 and potato PI-2. We found no QTLs in 2012. This may be related to the physiological state of the propagated seed tubers that were used as starting material in the field trial of 2012. Seed tubers should be propagated at least one cycle to carry out more reliable field trials as the physiological state of seed tubers may have an strong effect on plant development (Asiedu et al. 2003).

In this three-year study, a limited amount of overlap was found between QTLs over the years. This phenomenon is reflected by the low to poor correlations of the trait values between the years. QTLs on chromosome *2* (2014), *3* (2013) and *9* (2013) overlapped with the QTLs of the BLUEs of 2012–2014. The QTL on chromosome *5* was identified only in 2014. The low reproducibility of these QTLs may (partly) be attributed to the experimental design. The population was grown at three different locations over 3 years with one or two biological replicates. When dealing with a quantitative trait with a moderate level of heritability, such as protein content, the reproducibility of QTLs may be improved by making use of more biological replicates to compensate for possible environmental effects such as heterogeneity of soil quality and possible differences in (nitrogen) fertilizer residues in the plots of the trial fields.

### Strategies for trait improvement

This study illustrates that the genetic architecture of protein content in tetraploid potato is quantitative and complex. The moderate level of trait heritability in this study indicates that a substantial proportion of the trait variance can potentially be ascribed to heritable factors (QTLs). In the Altus × Colomba population we estimated a moderate level of broad sense trait heritability between 40 and 74% and identified potential naive QTLs on chromosomes *2*, *3*, *5* and *9*.

The cumulative variance explained by the naive QTLs identified within 2013, 2014 and BLUEs of 2012–2014 accounted between 11.9 and 17.2% of the total phenotypic variation. These proportions of variance closely resemble the cumulative variance explained by the QTLs together. Thus it can be postulated that these QTLs exert additive effects on protein content. Therefore, protein content in potato can be improved by fixating alleles with positive effects that underlie these QTLs in gene pools for breeding. However, in this specific mapping population it is evident that a large part of the trait heritability is not explained by the identified genetic factors alone. Further research is needed to elucidate these factors (e.g. minor effects QTLs and genotype-by-environment interactions) that may contribute towards this phenomenon.

To improve protein content in potato by means of molecular breeding, a more complete and comprehensive understanding of the genetic architecture and regulation of the trait is needed. A full overview of all protein content related QTLs—and their potential pleiotropic effects—that are present in relevant gene pools (e.g. the starch potato genepool) and uncultivated material provide a better understanding of the trait that is necessary for conscious decision-making in breeding programs. Further genetic studies, that include genome-wide association studies (GWAS) of variety panels and additional QTL analyses—both bi-parental and di-allel—are necessary for generating further insights into this economically and environmentally relevant trait. To take potential pleiotropic effects into account, these panels and populations should preferable include a similar maturity index, a uniform onset of tuber formation, fresh tuber yield, tuber under-water weight and overall yield stability across different environmental conditions. An alternative and classical strategy for trait improvement is the long term-selection on a trait of interest. Long-term selection programs have shown that this approach can be highly effective for increasing protein content in maize (Dudley 2007). This selection strategy allows the stacking and fixation of alleles with positive effects in the gene-pool for the trait of interest. For potato, this approach should without doubt also include the selection of other important potato traits such as pathogen resistances (e.g. late blight and potato cyst nematodes) and quality parameters (e.g. glycoalkaloid content). However, potential trade-offs should also be considered in the development of protein-rich potato varieties. This may include the use of heavier inputs of (mineral) nitrogen fertilizers that for a large part may be lost due to run-off and leaching into groundwater, thus causing environmental pollution. Therefore the question remains whether the nitrogen-use efficiency of protein-rich potato varieties will be improved, especially in the light of increasing attention for environmentally sustainable agriculture.

## Electronic supplementary material

Below is the link to the electronic supplementary material.

Supplementary material 1 (TIFF 165 kb)

Supplementary material 2 (TIFF 165 kb)

Supplementary material 3 (TIFF 166 kb)

Supplementary material 4 (TIFF 163 kb)

Supplementary material 5 (TIFF 165 kb)

Supplementary material 6 (TIFF 165 kb)

Supplementary material 7 (TIFF 171 kb)

Supplementary material 8 (TIFF 172 kb)

Supplementary material 9 (TIFF 171 kb)

Supplementary material 10 (TIFF 172 kb)

Supplementary material 11 (TIFF 172 kb)

Supplementary material 12 (TIFF 172 kb)

Supplementary material 13 (CSV 1099 kb)
