# Supplementary figures and images for: Multi-allelic QTL analysis of protein content in a bi-parental population of cultivated tetraploid potato
Source: Euphytica. 2019 Jan 8;215(2):14. doi: 10.1007/s10681-018-2331-z (PMC6390886; doi:10.1007/s10681-018-2331-z)

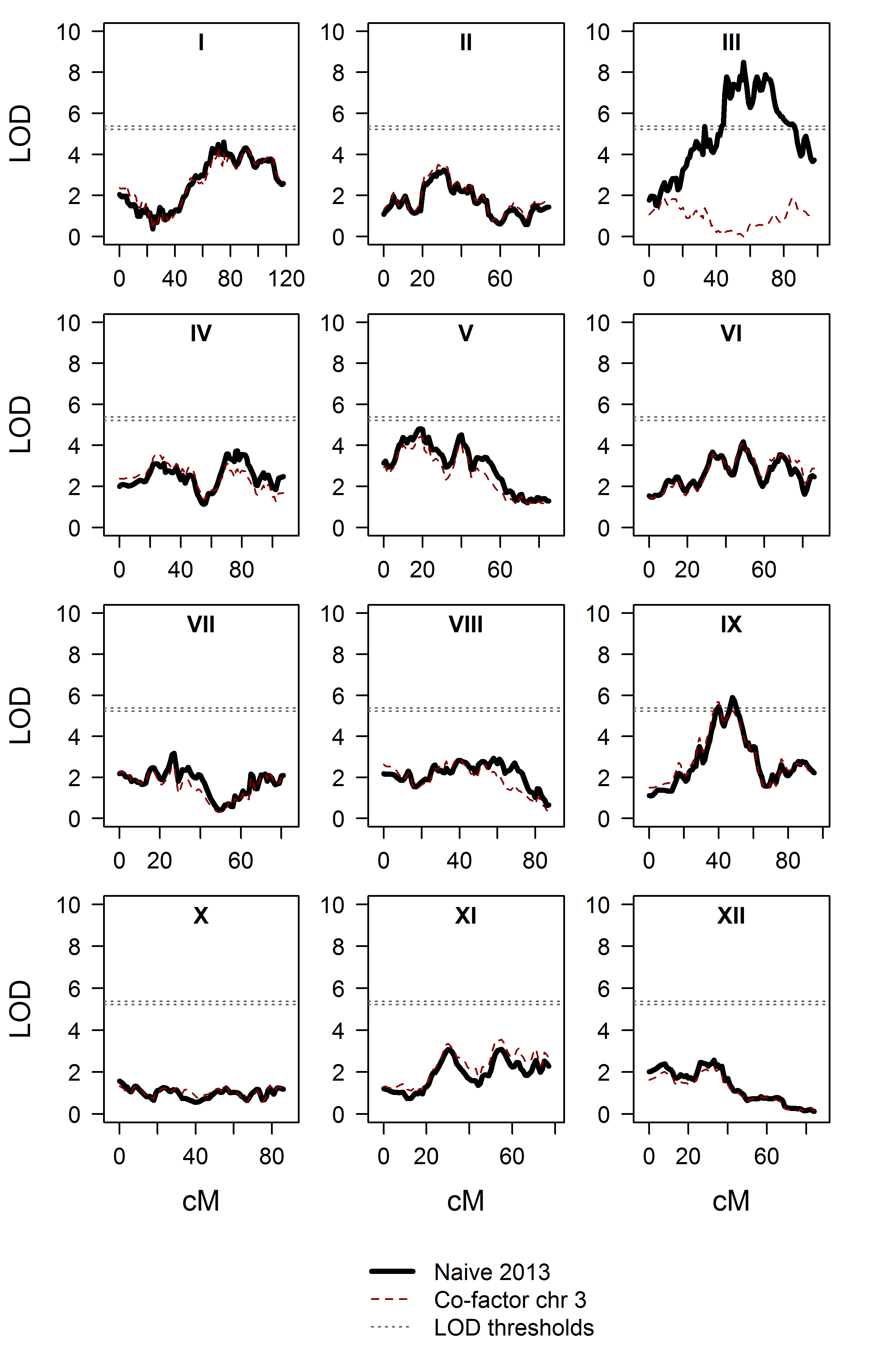

Supplement: Supplementary file 1 — Supplementary material 1 (TIFF 165 kb) [file 10681_2018_2331_MOESM1_ESM.tif]

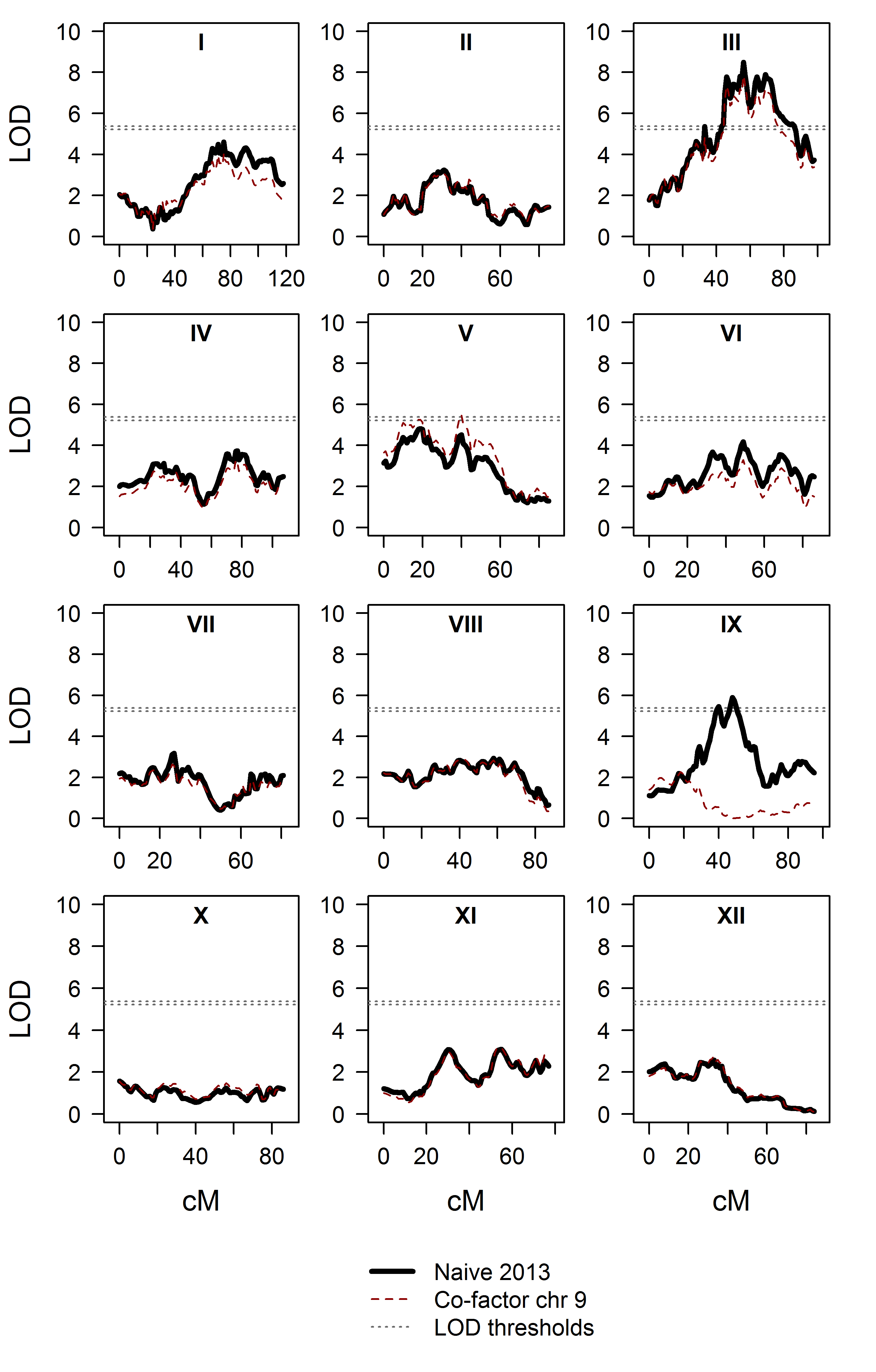

Supplement: Supplementary file 2 — Supplementary material 2 (TIFF 165 kb) [file 10681_2018_2331_MOESM2_ESM.tif]

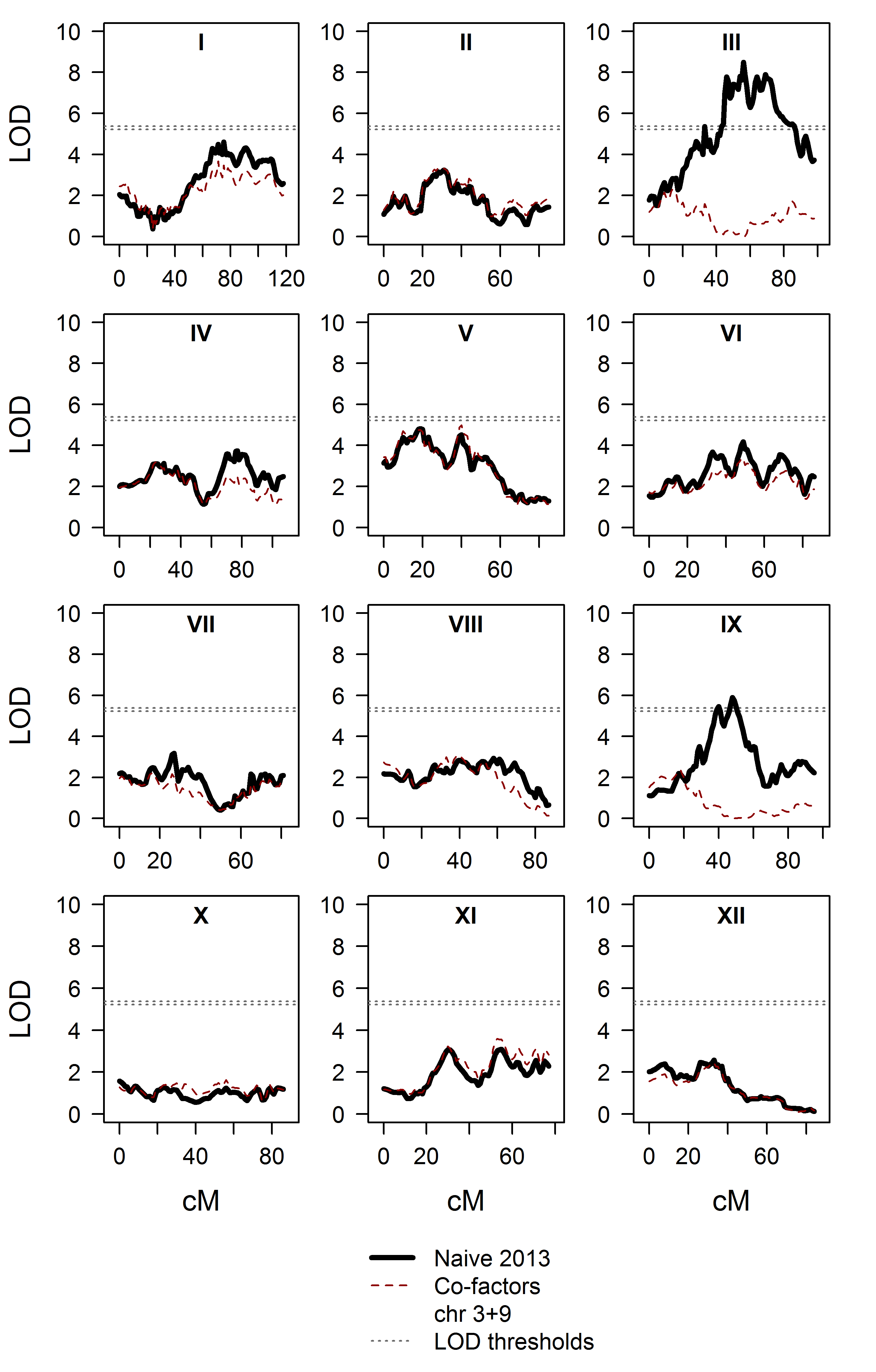

Supplement: Supplementary file 3 — Supplementary material 3 (TIFF 166 kb) [file 10681_2018_2331_MOESM3_ESM.tif]

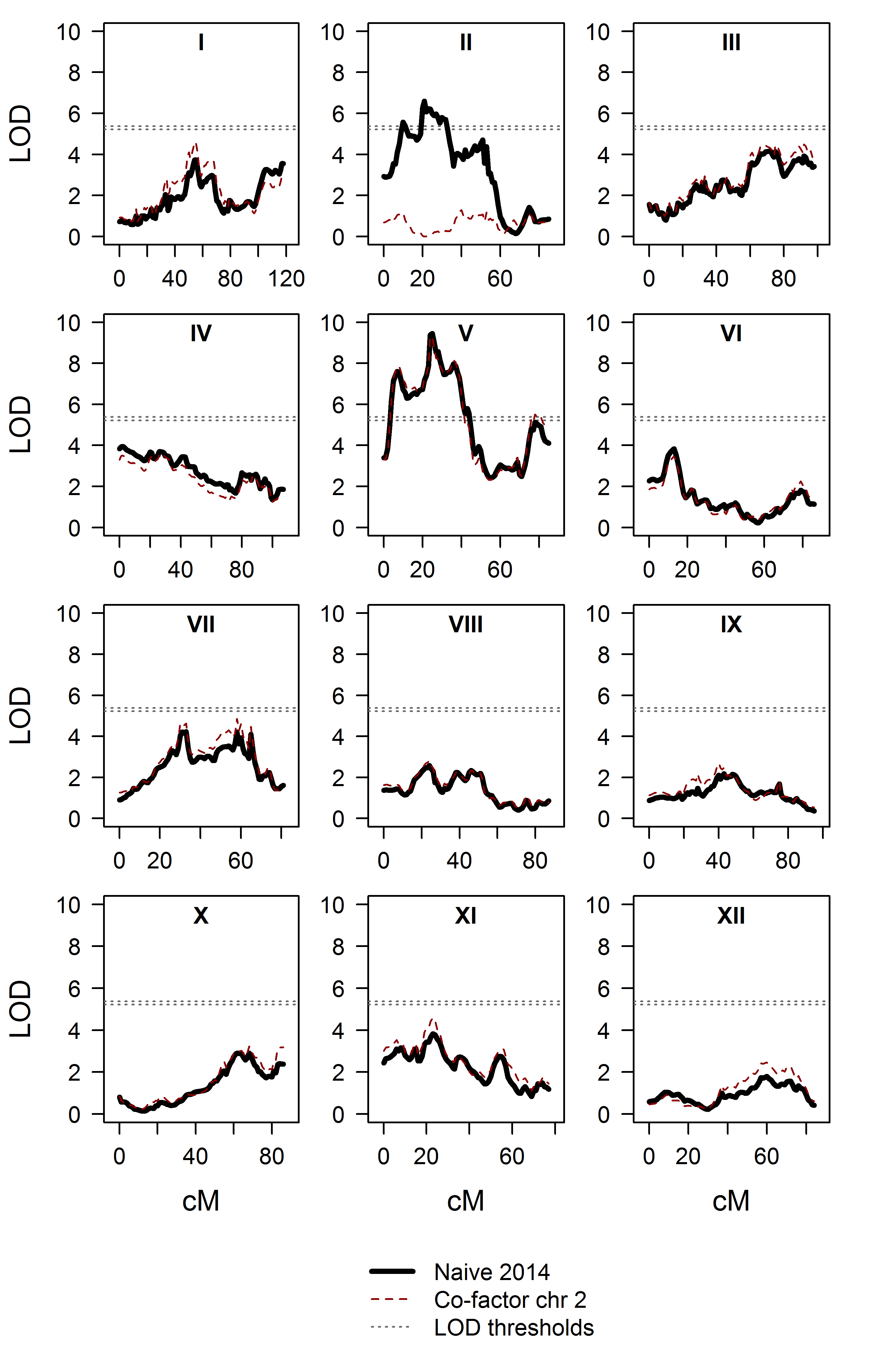

Supplement: Supplementary file 4 — Supplementary material 4 (TIFF 163 kb) [file 10681_2018_2331_MOESM4_ESM.tif]

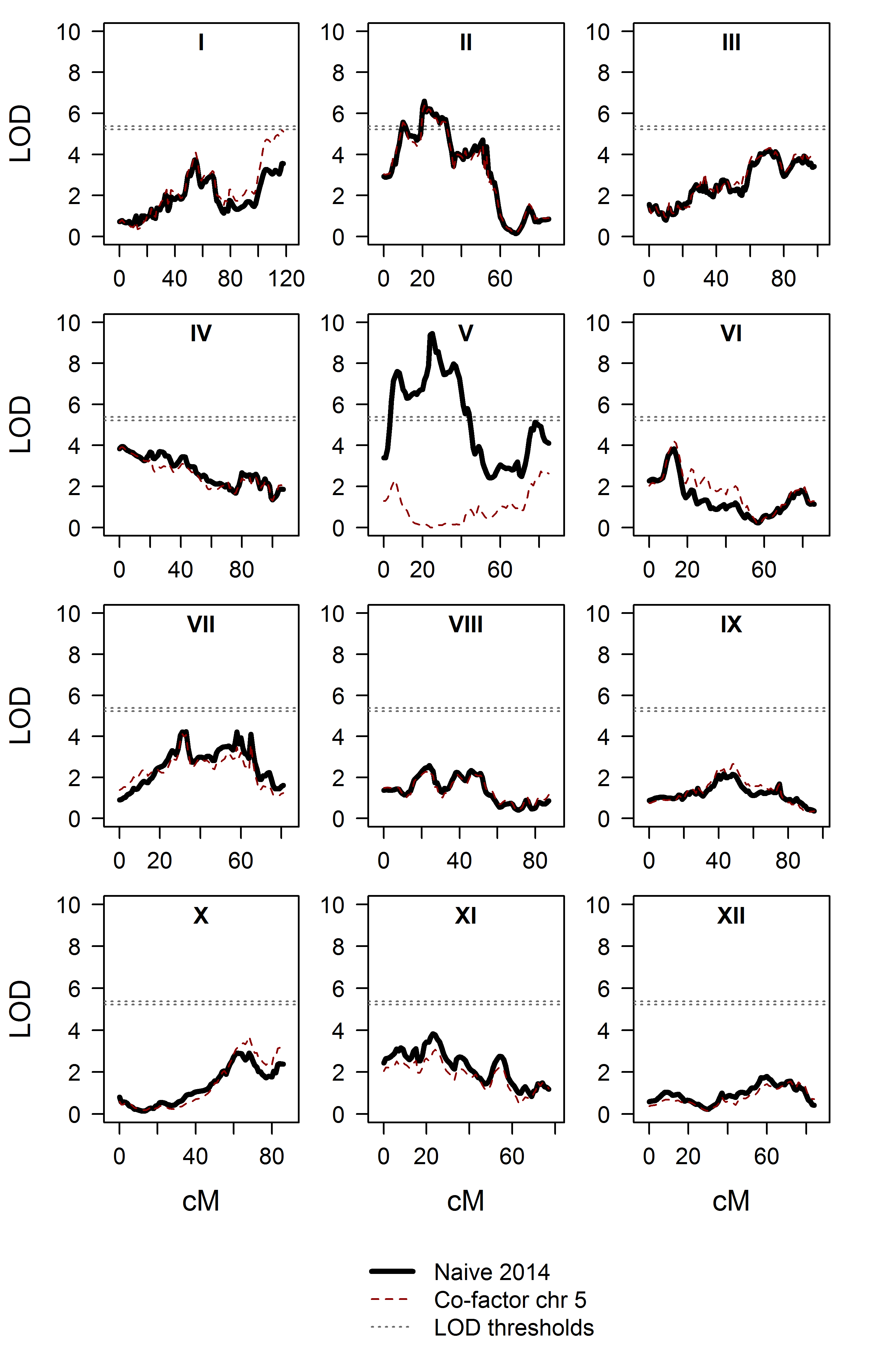

Supplement: Supplementary file 5 — Supplementary material 5 (TIFF 165 kb) [file 10681_2018_2331_MOESM5_ESM.tif]

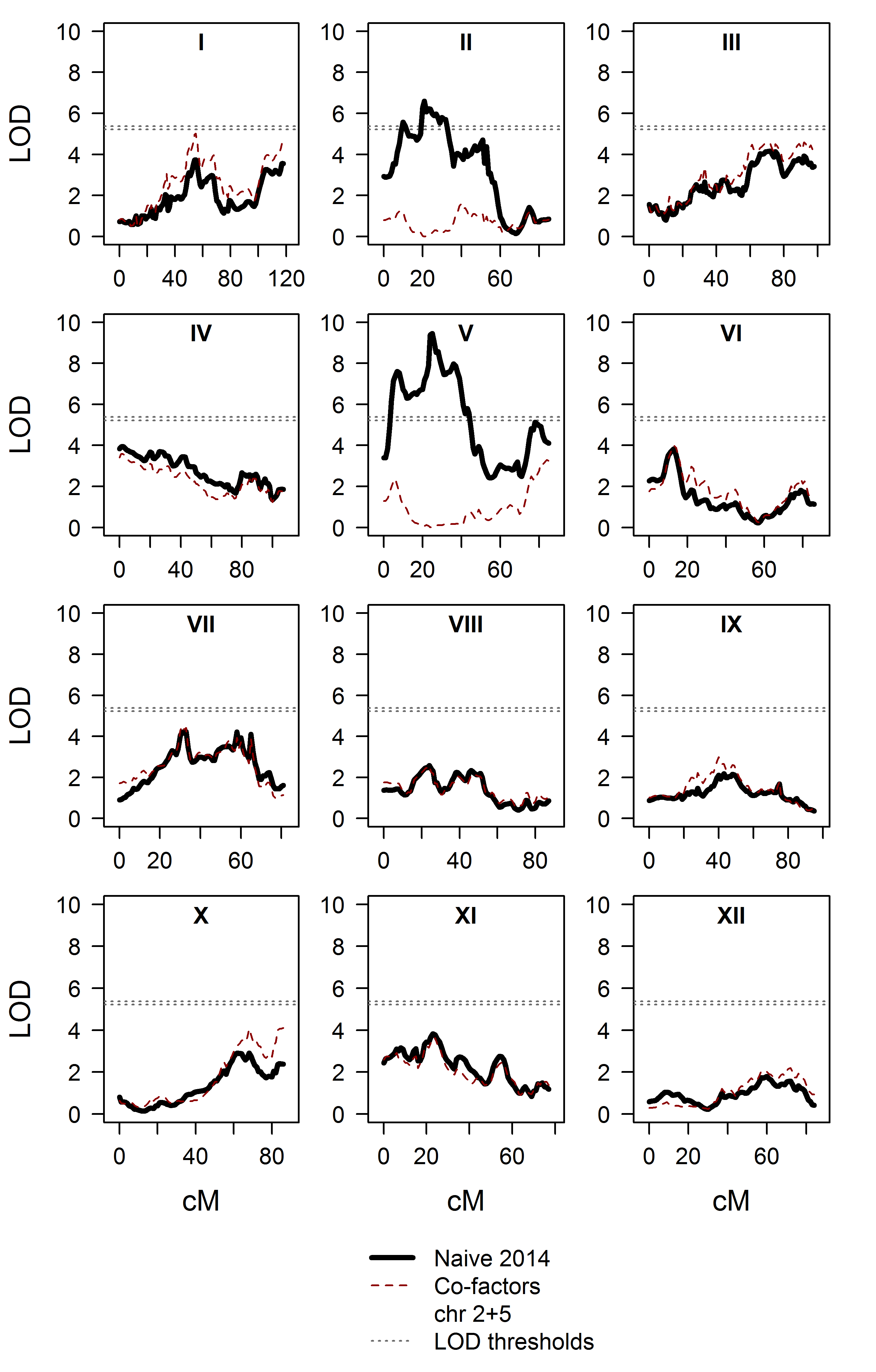

Supplement: Supplementary file 6 — Supplementary material 6 (TIFF 165 kb) [file 10681_2018_2331_MOESM6_ESM.tif]

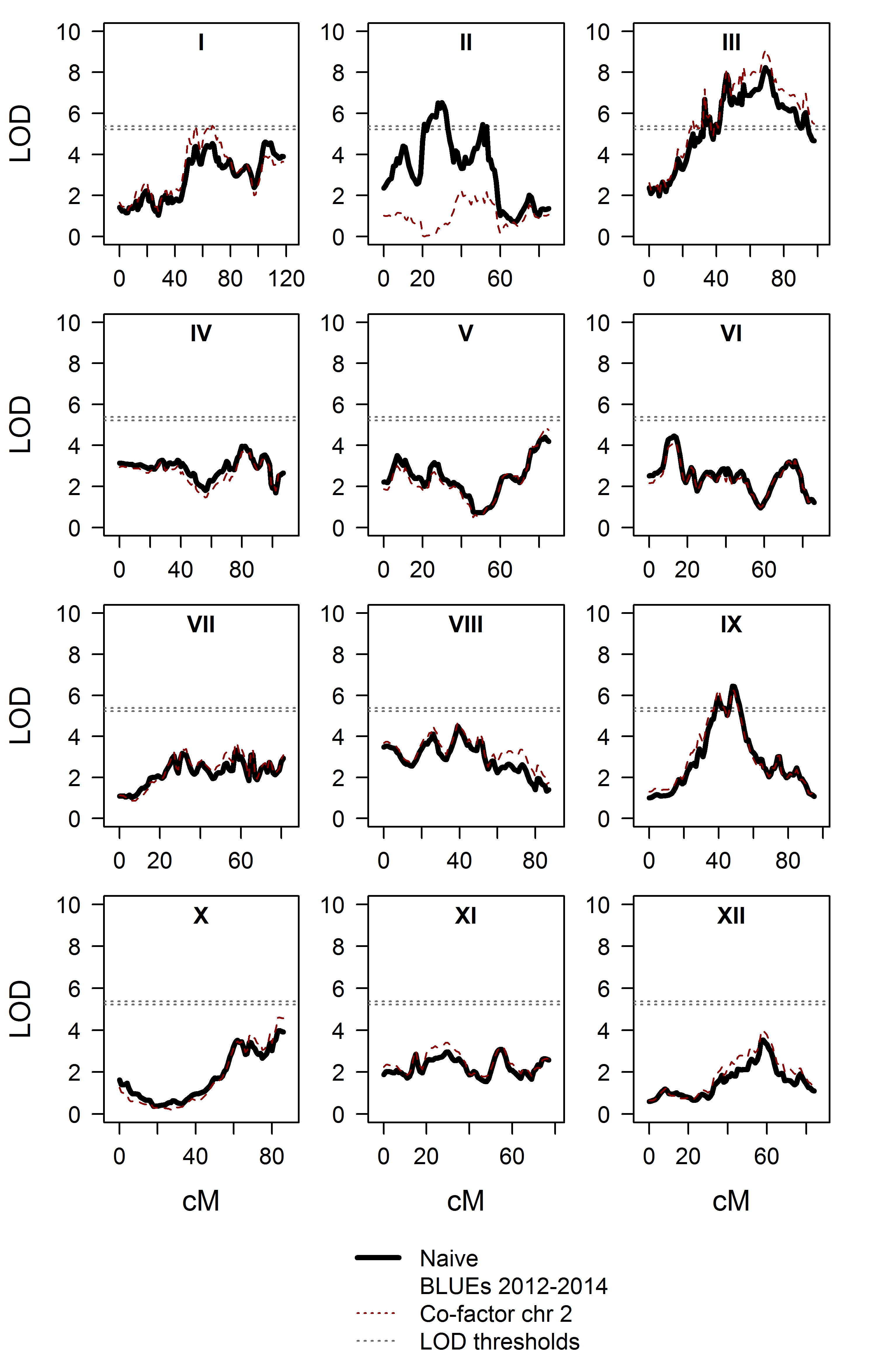

Supplement: Supplementary file 7 — Supplementary material 7 (TIFF 171 kb) [file 10681_2018_2331_MOESM7_ESM.tif]

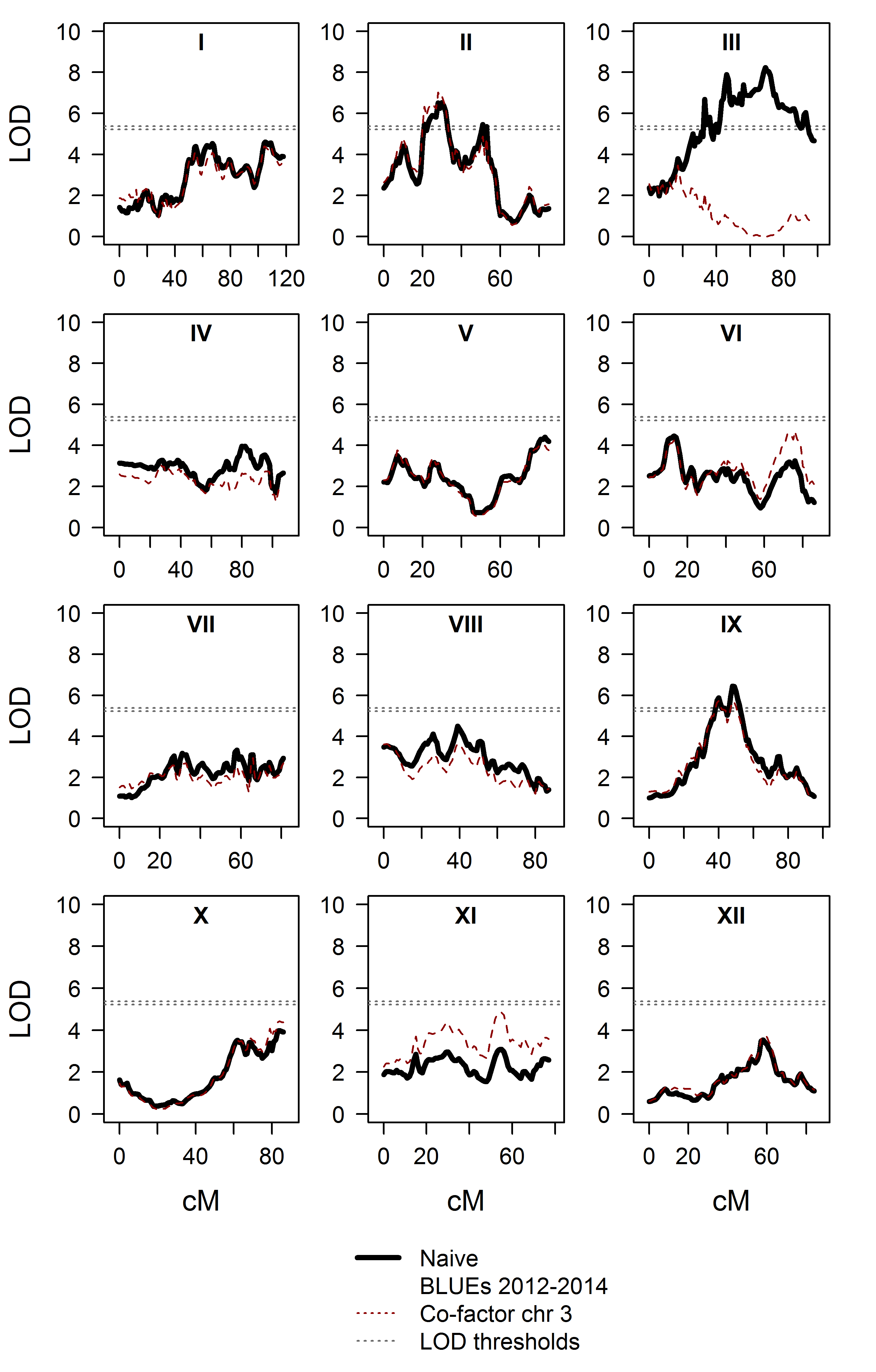

Supplement: Supplementary file 8 — Supplementary material 8 (TIFF 172 kb) [file 10681_2018_2331_MOESM8_ESM.tif]

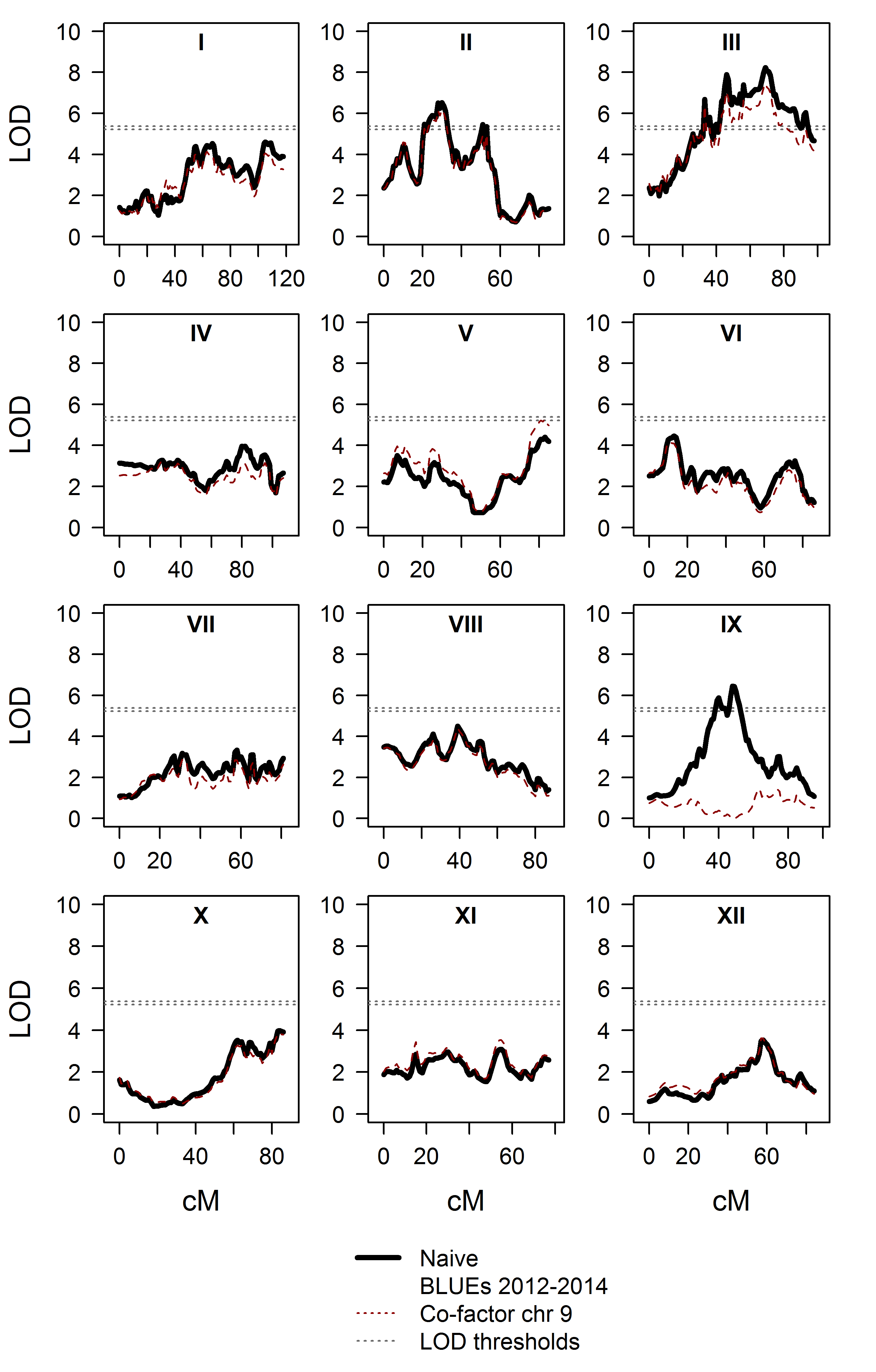

Supplement: Supplementary file 9 — Supplementary material 9 (TIFF 171 kb) [file 10681_2018_2331_MOESM9_ESM.tif]

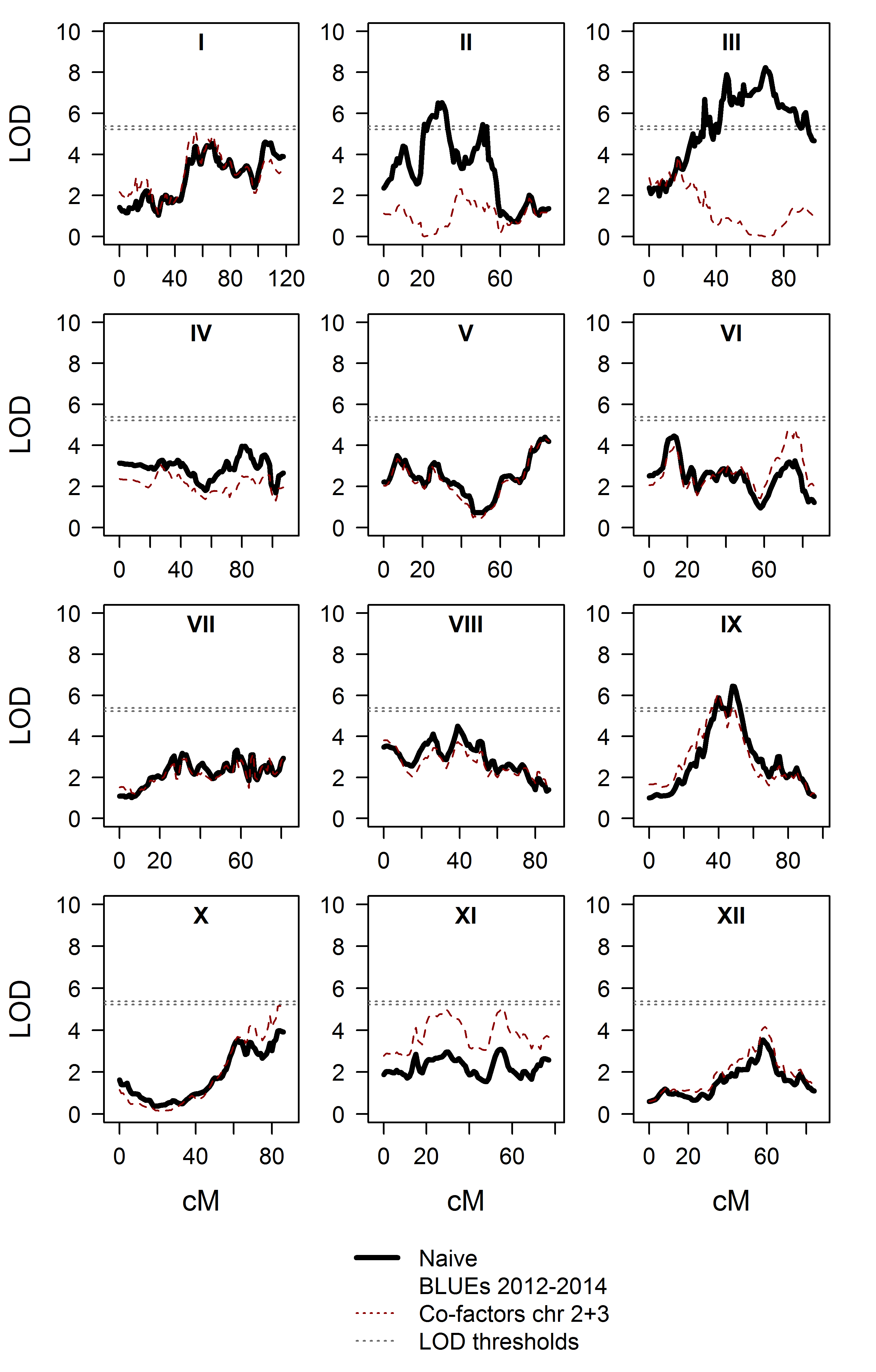

Supplement: Supplementary file 10 — Supplementary material 10 (TIFF 172 kb) [file 10681_2018_2331_MOESM10_ESM.tif]

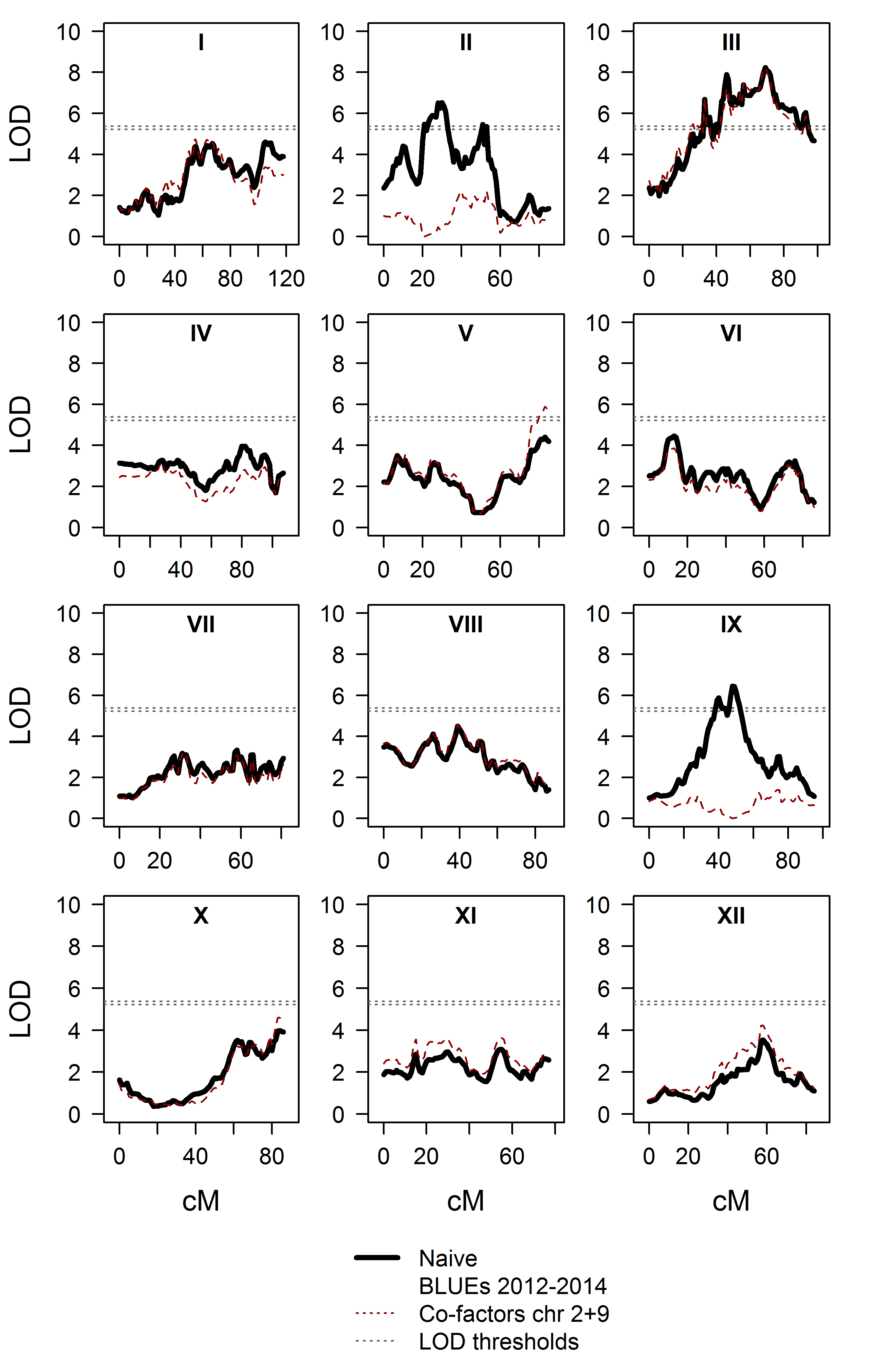

Supplement: Supplementary file 11 — Supplementary material 11 (TIFF 172 kb) [file 10681_2018_2331_MOESM11_ESM.tif]

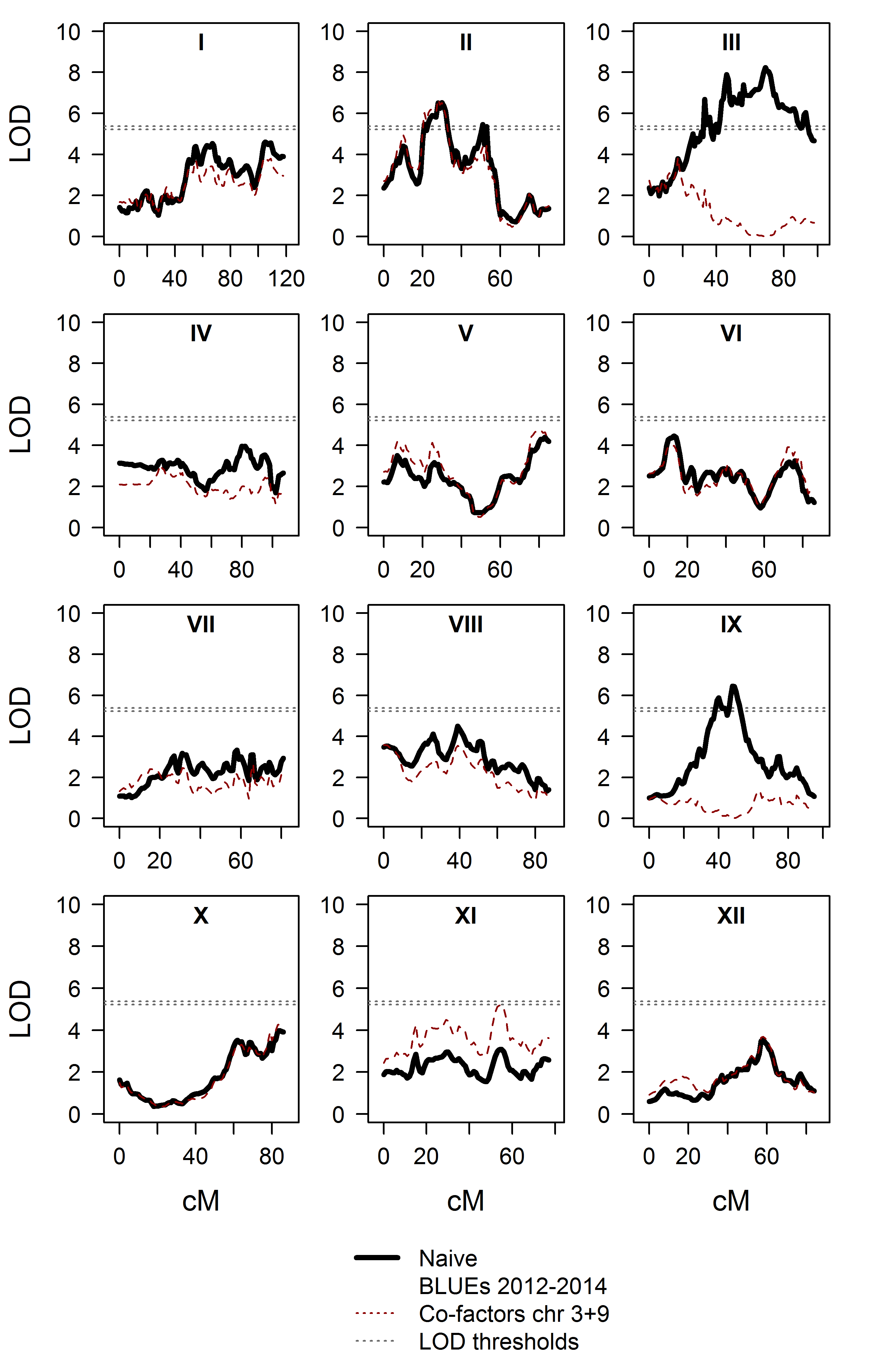

Supplement: Supplementary file 12 — Supplementary material 12 (TIFF 172 kb) [file 10681_2018_2331_MOESM12_ESM.tif]
